# A PD-L1-targeting chimeric switch receptor enhances efficacy of CAR-T cell for pleural and peritoneal metastasis

**DOI:** 10.1038/s41392-022-01198-2

**Published:** 2022-11-19

**Authors:** Qizhi Ma, Xia He, Benxia Zhang, Fuchun Guo, Xuejin Ou, Qiyu Yang, Pei Shu, Yue Chen, Kai Li, Ge Gao, Yajuan Zhu, Diyuan Qin, Jie Tang, Xiaoyu Li, Meng Jing, Jian Zhao, Zeming Mo, Ning Liu, Yao Zeng, Kexun Zhou, Mingyang Feng, Weiting Liao, Wanting Lei, Qiu Li, Dan Li, Yongsheng Wang

**Affiliations:** 1 Thoracic Oncology Ward, Cancer Center, and State Key Laboratory of Biotherapy, West China Hospital, Sichuan University, Chengdu, China; 2 State Key Laboratory of Biotherapy, West China Hospital, Sichuan University, Chengdu, China; 3 Cancer Center, West China Hospital, Sichuan University, Chengdu, China; 4 Institute of Respiratory Health, Frontiers Science Center for Disease-related Molecular Network, and Precision Medicine Research Center, Precision Medicine Key Laboratory of Sichuan Province, West China Hospital, Sichuan University, Chengdu, China

**Keywords:** Drug development, Tumour immunology, Metastasis

## Abstract

Pleural and peritoneal metastasis accompanied by malignant pleural effusion (MPE) or malignant ascites (MA) is frequent in patients with advanced solid tumors that originate from the lung, breast, gastrointestinal tract and ovary. Regional delivery of CAR-T cells represents a new strategy to control tumor dissemination in serous cavities. However, malignant effusions constitute an immune-suppressive environment that potentially induces CAR-T cell dysfunction. Here, we demonstrated that the anti-tumor cytotoxicity of conventional 2nd-generation CAR-T cells was significantly inhibited by both the cellular and non-cellular components of MPE/MA, which was primarily attributed to impaired CAR-T cell proliferation and cytokine production in MPE/MA environment. Interestingly, we found that PD-L1 was widely expressed on freshly-isolated MPE/MA cells. Based on this feature, a novel PD-L1-targeting chimeric switch receptor (PD-L1.BB CSR) was designed, which can bind to PD-L1, switching the inhibitory signal into an additional 4-1BB signal. When co-expressed with a 2nd-generation CAR, PD-L1.BB CSR-modified CAR-T cells displayed superior fitness and enhanced functions in both culture medium and MPE/MA environment, causing rapid and durable eradication of pleural and peritoneal metastatic tumors in xenograft models. Further investigations revealed elevated expressions of T-cell activation, proliferation, and cytotoxicity-related genes, and we confirmed that PD-L1 scFv and 4-1BB intracellular domain, the two important components of PD-L1.BB CSR, were both necessary for the functional improvements of CAR-T cells. Overall, our study shed light on the clinical application of PD-L1.BB CSR-modified dual-targeting CAR-T cells. Based on this study, a phase I clinical trial was initiated in patients with pleural or peritoneal metastasis (NCT04684459).

## Introduction

Pleural and peritoneal metastasis accompanied by malignant pleural effusion (MPE) or malignant ascites (MA) is a frequent and disabling complication in patients with advanced-stage cancers that originate from the lung, breast, gastrointestinal tract and ovary, which predicts a rapidly-deteriorating quality of life and extremely poor prognosis. With no cure for MPE/MA, efforts have been focused on symptomatic management rather than tumor control or prolongation of survival.^1–3^ Under current guidelines, limited therapeutic effects have been achieved by strategies including pleurodesis, indwelling catheters, peritoneovenous shunts and intrapleural/intraperitoneal chemotherapy. Moreover, patients frequently suffer from adverse events such as persistent pain, dyspnea, abdominal distention, peritoneal inflammation or/and ileus, with a median survival of less than 12 months.^1,2,4^ Since MPE/MA is triggered by tumor dissemination to pleural and abdominal cavities,^1,5^ only palliative management is insufficient for turning off effusion formation, and the ultimate therapeutic goal should be focused on aggressive tumor control.

In the last few years, regional administration of cytotoxic and immunotherapeutic agents has become a promising treatment modality for pleural and peritoneal metastasis.^1–3,6,7^ Notably, a recent phase I clinical trial has reported promising anti-tumor efficacy and good tolerance of regionally delivered mesothelin-targeted chimeric antigen receptor (CAR)-T cells in combination with an anti-programmed death receptor-1(PD-1) agent pembrolizumab in patients with malignant pleural diseases.^7^ On the other hand, despite the remarkable success achieved in hematological malignancies, CAR-T cells have been much less effective in solid tumors, and this is largely due to the low specificity and high heterogeneity of tumor antigens, poor T-cell infiltration into tumors, as well as the immune-suppressive tumor microenvironment that leads to T-cell exhaustion and dysfunction.^8^ Regional delivery of CAR-T cells circumvents the first two obstacles with extended antigens availability and direct access to tumor site, and meanwhile exhibits favorable safety and improved anti-tumor response.^7,9,10^ More importantly, postoperative recurrences in patients with certain types of cancer are commonly confined to pleural or peritoneal cavities, such as malignant mesothelioma, lung cancer, gastric cancer and especially ovarian cancer.^11–14^ Therefore, local infusion of CAR-T cells may provide a promising option for these patients.

Nevertheless, when CAR-T cells are infused into pleural or peritoneal cavities to treat metastatic tumors, they have to accommodate themselves to a complex immune-inhibitory environment, and can frequently encounter malignant effusions, since MPE/MA is difficult to be drained completely and contains high levels of immunosuppressive cytokines and cells.^15–19^ Furthermore, several studies found that tumor cells and immune cells could be induced by MPE/MA to overexpress programmed death-ligand 1 (PD-L1), which is closely associated with poor prognosis.^18,20,21^ In addition, retrospective studies demonstrated that the efficacy of PD-L1 inhibitor was significantly impaired by MPE/MA, resulting in shortened PFS and OS in non-small cell lung cancer (NSCLC) with PD-L1 ≥ 1%, as well as dMMR/MSI-H metastatic colorectal and gastric cancers.^22,23^ The poor outcomes can be attributed to MPE/MA-induced effector T-cell dysfunction.^5,18,24^ Together, these reports highlighted the necessity for environment-based CAR-T cell optimization.

Here, we reported a novel PD-L1-targeting chimeric switch receptor (PD-L1.BB CSR), which can bind to PD-L1 antigen in MPE/MA environment, switching the inhibitory signal into a 4-1BB signal. By simultaneously expressed on T-cells with a 2nd-generation tumor-associated antigen (TAA)-specific CAR, PD-L1.BB CSR-modified dual-targeting CAR-T cells exhibited enhanced fitness and functions in patient-derived MPE/MA. More importantly, regional delivery of the dual-targeting CAR-T cells caused rapid and durable eradication of pleural and peritoneal metastatic tumors in xenograft models. Our study shed light on the clinical application of PD-L1.BB CSR-modified dual-targeting CAR-T cells for pleural and peritoneal metastatic tumor treatment. Based on this study, a phase I clinical trial was initiated in patients with pleural or peritoneal metastasis.

## Results

### HER2.28ζ CAR-T cell functions are significantly impaired in MPE/MA

**Fig. 1:**
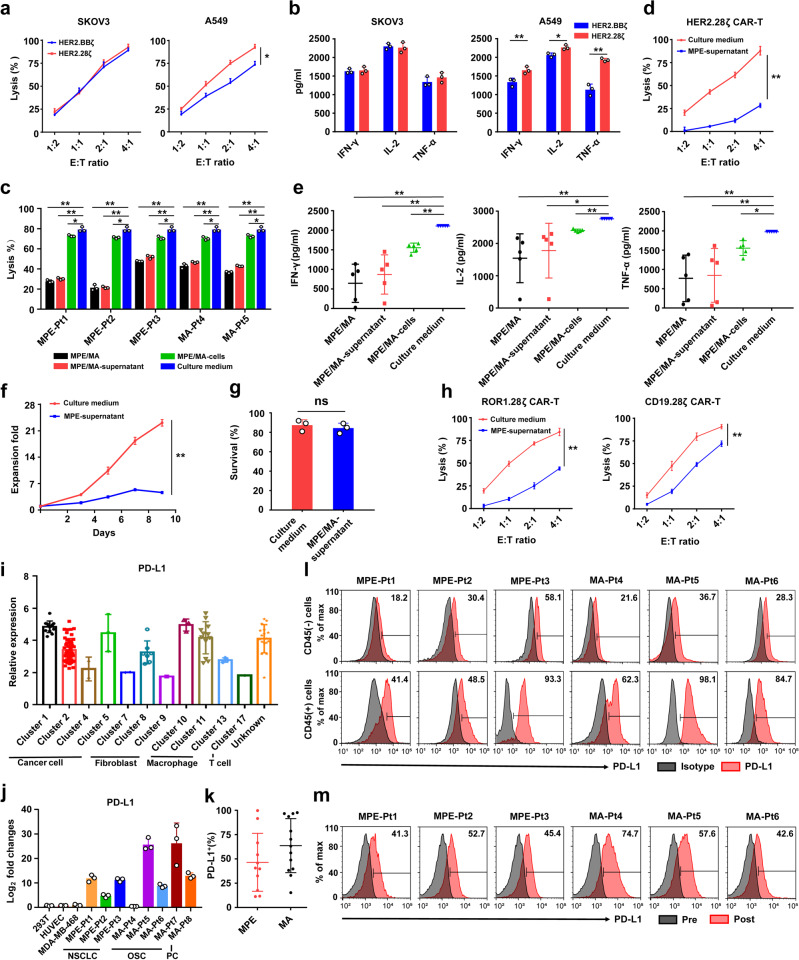
The functions of 2nd-generation CAR-T cells are significantly impaired in MPE/MA environment, and it is partially related to MPE/MA-induced PD-L1 expression. **a** The cytotoxicity of HER CAR-T cells (HER2.28ζ and HER2.BBζ) that incubated with SKOV3 or A549 cells for 24 h in cytokine-free medium (culture medium) was analyzed by CCK-8 assay. **b** Summary of IFN-γ, IL-2 and TNF-α release by HER2.28ζ and HER2.BBζ CAR-T cells after 24 h of co-incubation with SKOV3 or A549 (E: T = 2: 1) as measured by ELISA assay. **c** The cytotoxicity of HER2.28ζ CAR-T cells was analyzed by CCK-8 assay, after 48 h of co-culture with SKOV3 cells (E: T = 2: 1) in different components of patient-derived MPE or MA samples (Supplementary Table. S1). **d** The cytotoxicity of HER2.28ζ CAR-T cells was analyzed by CCK-8 assay, after 24 h of co-culture with SKOV3 cells in culture medium or MPE-supernatant (Pt1, as Supplementary Table. S1). **e** The levels of cytokines (IFN-γ, IL-2 and TNF-α) in the supernatants from (**c**) were determined by ELISA. **f** The fold expansion of HER2.28ζ CAR-T cells were analyzed through FCM-counting beads, after stimulated by irradiated SKOV3 cells (E: T = 2: 1) in culture medium or MPE-supernatant (Pt1, as Supplementary Table. S1), and the portion of live CAR-T cells (Annexin-V^-^/PI^-^) on the 3rd day after stimulation was shown in **g**. **h** The cytotoxicity of ROR1.28ζ and CD19.28ζ CAR-T cells were co-cultured with SKOV3 and Raji cells respectively in culture medium or MPE-supernatant (Pt1, as Supplementary Table. S1) for 24 h, and analyzed by CCK-8 assay. **i** The mRNA expression of PD-L1 in cancer cells, fibroblasts, macrophages and T cells of human ovarian cancer ascites analyzed by single-cell RNA-sequencing (scRNA-seq) data deposited in public Gene Expression Omnibus (GSE146026). **j** PD-L1 mRNA expression levels in MPE/MA-cells were shown as fold change to 293 T, HUVEC and MDA-MB-468 cells (as controls). **k** The statistical analysis of PD-L1 expression on clinically derived MPE/MA-cells (Supplementary Table. S1) was analyzed by flow cytometry. **l** Representative flow cytometry histogram of PD-L1 expression on CD45^-^ and CD45^+^ cells from MPE/MA samples (Supplementary Table. S1). **m** Representative flow cytometry histogram of PD-L1 expression on healthy donor-derived CD3^+^ T cells that cultured in MPE/MA-supernatant (Supplementary Table. S1) continuously for 7 days. (Pre: pre-culturing; Post: post-culturing) Pt, patient; MPE, malignant pleural effusion; MA, malignant ascites. *P*-values were determined by unpaired two-tailed t-test **a**, **b**, **d**, **f**, **g**, **h** or one-way ANOVA with Tukey’s multiple comparison test adjusted *P* value **c**, **e**. *P <0.05; **P <0.01; ns, not significant. Data show the mean ± SD from three independent experiments

To date, CD28 and 4-1BB are the two most commonly used co-stimulatory signals in CAR design. It has been reported that after activation, CD28 co-stimulated CAR-T cells released higher levels of effector cytokines including IL-2, IFN-γ, and TNF-α, as compared to 4-1BB co-stimulated counterparts, however, conflicting data exist concerning this perception.^25,26^ HER2 is expressed on most solid tumors that are prone to form malignant effusion after pleural or peritoneal dissemination, including the lung, breast, gastric and ovarian cancers. To select a better co-stimulatory signal for HER2 CAR-T cells to treat pleural and peritoneal metastatic tumors, T-cells from healthy donors were transfected by lentivirus to express either a HER2.28ζ or HER2.BBζ CAR. The antitumor activity of CAR-T cells was evaluated against HER2 high-expression SKOV3 ovarian cancer cells and HER2 moderate-expression A549 lung adenocarcinoma cells, respectively. When co-cultured with SKOV3 cells, the cytolytic activities were comparable between HER2.28ζ and HER2.BBζ CAR-T cells at various effector-to-target (E:T) ratios. However, when co-cultured with A549 cells, HER2.28ζ CAR-T cells showed significantly higher cytotoxicity compared to HER2.BBζ CAR-T cells, indicating superior anti-tumor activity of CD28 co-stimulated CAR-T cells (Fig. 1a). This disparity was further corroborated by the significantly higher IFN-γ, IL-2 and TNF-α release of HER2.28ζ CAR-T cells that co-incubated with A549 cells (Fig. 1b).

The dysfunction of effector T-cells in MPE/MA has been reported in previous studies.^5,18,24^ As HER2.28ζ CAR-T cells exhibited superior anti-tumor cytotoxicity than HER2.BBζ CAR-T cells, we next investigated the cytotoxicity of HER2.28ζ CAR-T cells that cultured in different components of MPE/MA samples from clinical patients (Supplementary Table. S1). We found that when cultured in the whole MPE/MA, MPE/MA-supernatant or MPE/MA-cells, HER2.28ζ CAR-T cell-mediated tumor cell killing was all significantly inhibited. And compared to MPE/MA-cells, MPE/MA-supernatant showed significantly stronger inhibition (Fig. 1c). To exclude the potential influence of target cell number, we further tested MPE/MA-mediated inhibition on CAR-T cytotoxicity at different E:T ratios in the MPE-supernatant of lung adenocarcinoma patient (Pt1). The result showed that HER2.28ζ CAR-T cell-mediated cytotoxicity to SKOV3 cells was significantly inhibited by over 50% at each E: T ratio (Fig. 1d). To further investigate the potential mechanism, CAR-T cell cytokine secretion, proliferation and apoptosis were assessed subsequently. As a result, a significant reduction of IFN-γ, IL-2 and TNF-α released by HER2.28ζ CAR-T cells was detected when co-cultured with SKOV3 cells in different components of MPE/MA (Fig. 1e).

In addition, HER2.28ζ CAR-T cells were CFSE labeled and incubated with 12 Gy-irradiated SKOV3 cells in MPE-supernatant (Pt1). Flow cytometry detection showed that CAR-T cell proliferation was prominently reduced (Fig. 1f). In contrast, no significant differences were observed for apoptotic CAR-T cells (Fig. 1g). More importantly, MPE-supernatant mediated functional inhibition was also observed in T-cells expressing ROR1.28ζ or CD19.28ζ, which demonstrated that the CAR-T cell suppression in MPE/MA environment was target-antigen independent (Fig. 1h). Overall, these evidences revealed that the 2nd-generation CAR gene-edited T cells were functionally inhibited in MPE/MA environment, and which was ascribed to impaired cytokine release and proliferation.

### PD-L1 is an ideal target for signal optimization of CAR-T cells

As shown above, MPE/MA-cells significantly impaired the anti-tumor cytotoxicity of 2nd-generation CAR-T cells, although the inhibition was weaker than MPE/MA-supernatant. Accumulating evidence has shown that MPE/MA induces PD-L1 expression on both tumor cells and immune cells, and simultaneously exacerbates PD-L1-mediated T-cell dysfunction.^18,20,21^ To investigate PD-L1 expression in malignant effusions, firstly, cells from human ovarian cancer ascites were analyzed, using public single-cell RNA-sequencing (scRNA-seq) data set (GSE146026) downloaded from Gene Expression Omnibus (GEO). The results showed that PD-L1 mRNA expression was significantly up-regulated in tumor cells, fibroblasts, macrophages and T-cells in ovarian cancer ascites (Fig. 1i). This was further validated by upregulated PD-L1 mRNA and protein expressions in MPE/MA-cells that harvested from patients with NSCLC, ovarian serous cystadenocarcinoma (OSC) and pancreatic cancer (PC). The results of Quantitative Real-Time PCR (qRT-PCR) showed that in the majority of MPE/MA samples, PD-L1 mRNA expression was higher than the average level of 293 T, HUVEC and MDA-MB-468 (Fig. 1j). Furthermore, flow cytometry found that PD-L1 was expressed on most MPE/MA-cells (Fig. 1k), especially on CD45^+^ leukocytes, such as T cells, B cells, myeloid-derived suppressor cells (MDSCs) and tumor‐associated macrophages (Fig. 1l and Supplementary Fig. S1a, b). To further confirm the findings, CD3^+^ T cells of healthy donors were cultured consecutively for 7 days in various patient-derived MPE/MA-supernatant. And as expected, remarkably upregulated PD-L1 expression was detected on all the T-cells (Fig. 1m).

Based on the above findings, we concluded that in addition to the direct functional inhibition of T-cell cytokine secretion and proliferation, MPE/MA could potentially impair CAR-T cell activities via PD-1/PD-L1 axis, by upregulating PD-L1 expression on tumor and non-tumor cells. Therefore, it is necessary to optimize CAR-T cell functions to overcome MPE/MA-mediated immune inhibition. Since PD-L1 is frequently overexpressed on solid tumor and immune cells, and involved in immune evasion and tumor dissemination,^18,20,21^ it can be an ideal target for CAR-T cell signal optimization.

### PD-L1.BB CSR engagement enhances the cytotoxicity of HER2.28ζ CAR-T cell in vitro

**Fig. 2:**
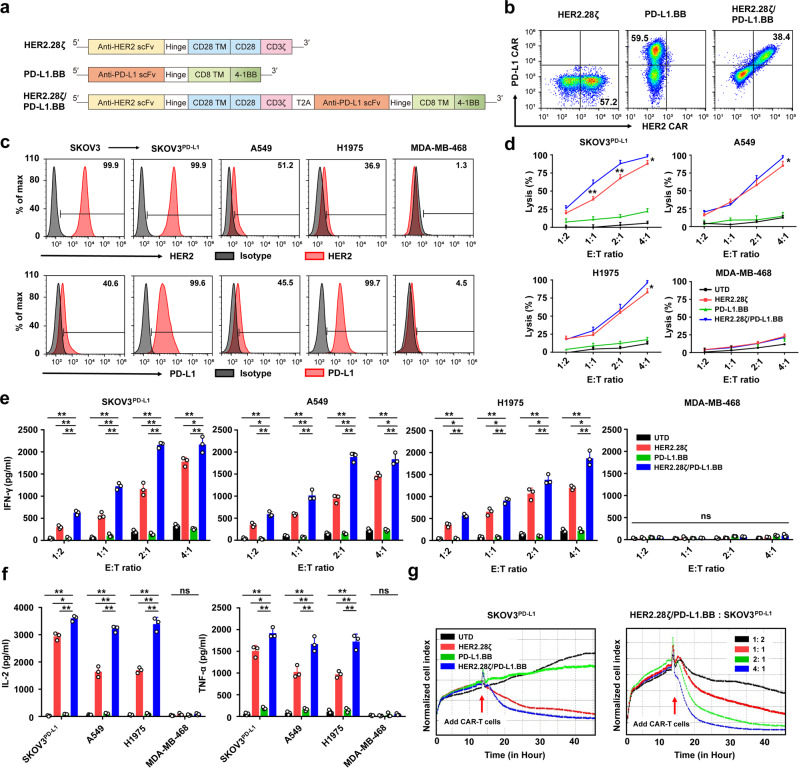
PD-L1.BB CSR engagement enhances the cytotoxicity and CAR activation-induced cytokine release of HER2.28ζ CAR-T cells. **a** Schematic illustration of lentiviral vectors encoding HER2.28ζ, PD-L1.BB and HER2.28ζ/PD-L1.BB. **b** Representative flow cytometry plots of CAR expressions on healthy donor derived CD3^+^ T cells. **c** Representative flow cytometry histograms of HER2 and PD-L1 expressions on various human tumor cell lines. **d** The cytotoxicities of CAR-T cells were analyzed by CCK-8 assay, after 24 h of co-culture with HER2^+^PD-L1^+^ (SKOV3^PD-L1^, A549 or H1975) or HER2^-^PD-L1^-^ (MDA-MB-468) cancer cells at various E:T ratios, and IFN-γ **e**, IL-2, and TNF-α **f** released by CAR-T cells (E: T = 2: 1) in the culture supernatant were determined by ELISA. **g** Continuous cytotoxicity of CAR-T cells was assessed by an xCELLigene Real-Time Cell Analysis (RTCA) system. Left: Untransduced (UTD) T cells, HER2.28ζ, PD-L1.BB and HER2.28ζ/PD-L1.BB CAR-T cells that co-incubated with SKOV3^PD-L1^ at E: T of 2: 1. Right: HER2.28ζ/PD-L1.BB CAR-T cells that co-incubated with SKOV3^PD-L1^ at various E: T ratios. *P*-values were determined by one-way ANOVA with Tukey’s multiple comparison test adjusted *P* value **d**–**f**. *P <0.05, **P <0.01, ns, not significant. Data show the mean ± SD from three independent experiments

After antigen-induced activation, the cytokine release and proliferation of CAR-T cells are primarily dependent on the co-stimulatory signals.^26^ Therefore, we hypothesized that CAR-T cells equipped with enhanced co-stimulatory signal may rescue MPE/MA-mediated impaired CAR-T cell functions. The additional co-stimulatory domain can either be placed in tandem as the 3rd-generation CAR-T cells, or in an individual CAR as the dual-targeting CAR-T cells.^27^ Considering the prevalence of PD-L1 expression in MPE/MA environment, a PD-L1-targeting CSR (PD-L1.BB) was designed, which was composed of an extracellular high-affinity anti-PD-L1 scFv and an intracellular 4-1BB domain. The 4-1BB domain was introduced, because 4-1BB co-stimulated CAR-T has been reported to exhibit superior IL-2 and IFN-γ production, T-cell proliferation, as well as in vivo survival and persistence.^26,28–30^ Based on the design of PD-L1.BB CSR, it can bind to PD-L1 antigen, and switch the inhibitory signal into an additional 4-1BB co-stimulatory signal. When co-expressed with a HER2.28ζ CAR, the novel dual-targeting CAR-T cells independently provided both dual-antigen specificities and dual-co-stimulatory signals by targeting HER2 and PD-L1 (HER2.28ζ/PD-L1.BB CAR, Fig. 2a).

As assessed by flow cytometry, both HER2.28ζ CAR and PD-L1.BB CSR were efficiently expressed on T-cells from healthy donors (Fig. 2b and Supplementary Fig. S2a). Phenotypic analysis showed that the CD4^+^ and CD8^+^ T cell ratios were comparable between each group (Supplementary Fig. S2b), and no significant differences were observed in the percentage of central memory T cell (CD45RO^+^CCR7^+^, T_CM_) **(**Supplementary Fig. S2c). To evaluate whether PD-L1.BB CSR engagement enhances the cytotoxicity of HER2.28ζ CAR-T cells to PD-L1^+^ target cells, both expressions of HER2 and PD-L1 on different cancer cell lines were detected, and PD-L1 overexpression SKOV3 cells (SKOV3^PD-L1^) were generated **(**Fig. 2c). Then HER2.28ζ/PD-L1.BB, HER2.28ζ, PD-L1.BB CAR-T cells, or untransduced (UTD) T cells were co-cultured with different tumor cells (SKOV3^PD-L1^, A549, H1975, or MDA-MB-468) at various E: T ratios (1:2, 1:1, 2:1, and 4:1) for 24 h in a cytokine-free medium (culture medium). As compared to HER2.28ζ CAR-T cells, HER2.28ζ/PD-L1.BB CAR-T cells exhibited superior cytotoxicity against all the three HER2^+^/PD-L1^+^ tumor cells (SKOV3^PD-L1^, A549 and H1975), particularly when cultured with SKOV3^PD-L1^ cells that had the highest HER2 and PD-L1 expressions among all target tumor cells. In contrast, when co-cultured with HER2^-^/PD-L1^-^ MDA-MB-468 cells, no significant differences were observed between each group (Fig. 2d). Additionally, we observed a significant increase of cytokine release in the culture supernatant from HER2.28ζ/PD-L1.BB group, as compared to HER2.28ζ group (Fig. 2e, f). Furthermore, the superior cytotoxicity of HER2.28ζ/PD-L1.BB CAR-T cells were also monitored using the xCELLigence Real-Time Cell Analysis (RTCA) SP Instrument (Fig. 2g). Overall, these data showed that PD-L1.BB CSR engagement significantly enhanced the cytotoxicity and effector cytokine release of HER2.28ζ CAR-T cells in vitro.

### PD-L1.BB CSR engagement enhances HER2.28ζ CAR-T cell functions in MPE/MA

**Fig. 3:**
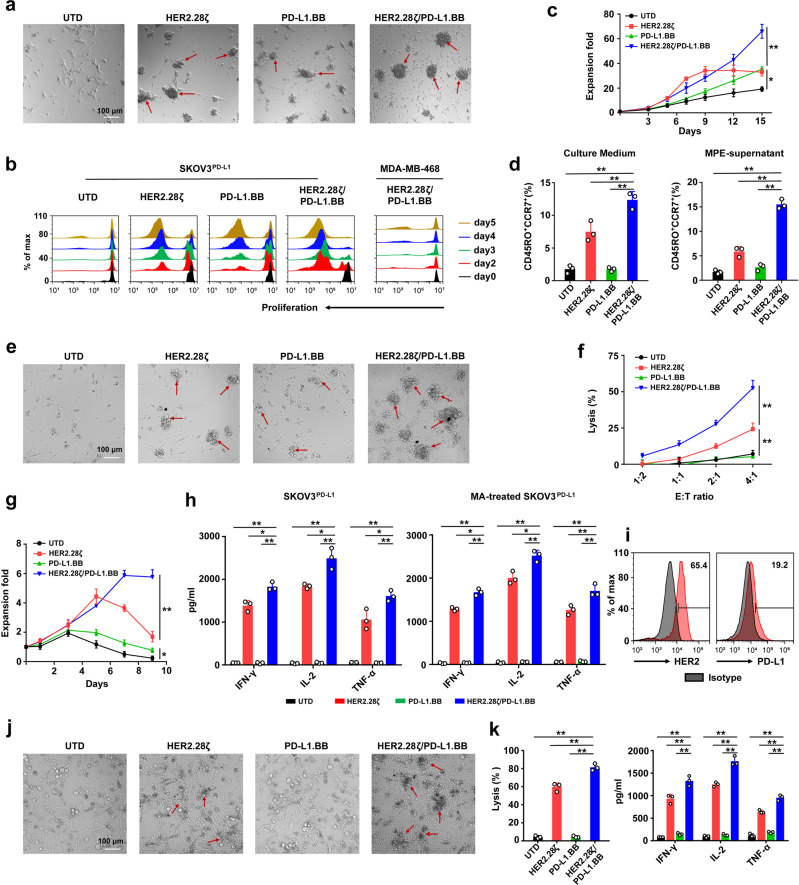
PD-L1.BB CSR engagement improves the functions of HER2.28ζ CAR-T cells and resists MPE/MA-mediated immunosuppression. **a** Representative microscopy images of CAR-T cell cluster formation after 24 h of co-culture with SKOV3^PD-L1^ cells (E: T = 2: 1) in culture medium. Magnification: 100×. **b** Proliferation of HER2.28ζ, PD-L1.BB and HER2.28ζ/PD-L1.BB CAR-T after activation. CFSE-labeled CAR-T stimulated by irradiated SKOV3^PD-L1^ or MDA-MB-468 cells (as control) on day 0 at E: T ratio of 2:1. CFSE dilution was measured by flow cytometry on days 2, 3, 4 and 5 after stimulation. **c** The fold expansion of CAR-T cells after stimulated by irradiated SKOV3^PD-L1^ cells (E: T = 2: 1) in culture medium was measured by FCM-counting beads. **d** The ratio of central memory T cells (CD45RO^+^CCR7^+^) in each group of CAR-T cells that cultured in culture medium or MPE (Pt1, as Supplementary Table. S1) was determined on day 3 post-stimulation by irradiated SKOV3^PD-L1^ cells (E: T = 2: 1). **e** Representative microscopy images of CAR-T cell cluster formation after 24 h of co-culture with SKOV3^PD-L1^ (E: T = 2: 1) in MPE-supernatant (Pt1, as Supplementary Table. S1). Magnification: 100×. **f** The cytotoxicity of CAR-T cells was analyzed by CCK-8 assay after 24 h of co-culture with SKOV3^PD-L1^ cells in MPE-supernatant (Pt1, as Supplementary Table. S1). **g** The fold expansion of CAR-T cells after stimulated by irradiated SKOV3^PD-L1^ cells (E: T = 2: 1) in MPE-supernatant (Pt1, as Supplementary Table. S1). **h** Summary of IFN-γ, IL-2 and TNF-α released by CAR-T cells after 24 h of co-culture with SKOV3^PD-L1^ or MA-treated SKOV3^PD-L1^ cells (E: T = 2: 1) in MA-supernatant (Pt4, as Supplementary Table. S1). MA-treated SKOV3^PD-L1^ cells: SKOV3^PD-L1^ cells that cultured continuously in MA-supernatant for 1 month. **i** Representative flow cytometry histograms of HER2 and PD-L1 expressions on MPE-isolated primary small-cell lung cancer (SCLC) cells (Pt10, as Supplementary Table. S1). **j** The representative microscopy images of CAR-T cell cluster formation after 48 h of co-incubation with primary SCLC cells (E: T = 2: 1) in MPE-supernatant from the same patient (Pt10, as Supplementary Table. S1). Magnification: 100×. **k** The primary SCLC cells viability was determined by CCK-8 assay. IFN-γ, IL-2 and TNF-α released by CAR-T cells in the supernatant were measured by ELISA. *P*-values were determined by one-way ANOVA with Tukey’s multiple comparison test adjusted *P* value (**c**, **d**, **f**, **g**, **h**, **k**). *P <0.05, **P <0.01, ns, not significant. Data show the mean ± SD from three independent experiments

To investigate whether PD-L1.BB CSR modification can improve the functions of HER2.28ζ CAR-T cell, firstly, HER2.28ζ/PD-L1.BB, HER2.28ζ, PD-L1.BB CAR-T cells, or UTD cells were co-cultured with SKOV3^PD-L1^ cells at E:T ratio of 2:1 in culture medium for 24 h. We observed the formation of obviously larger suspending cell clusters in HER2.28ζ/PD-L1.BB group in the meantime of efficient eradication of target cells, as compared to HER2.28ζ and PD-L1.BB groups, indicating increased proliferation activity of HER2.28ζ/PD-L1.BB CAR-T cells (Fig. 3a). To further confirm this phenomenon, CFSE-labeled CAR-T cells were cultured with 12 Gy-irradiated SKOV3^PD-L1^ cells, and then flow cytometry was used to measured CAR-T proliferation. Compared with T-cells expressing HER2.28ζ or PD-L1.BB alone, HER2.28ζ/PD-L1.BB CAR-T cells exhibited stronger and more persistent proliferation (Fig. 3b), which was further verified by the absolute T-cell counts (Fig. 3c). By contrast, no significant differences were observed in co-culture with irradiated MDA-MB-468 cells (Fig. 3b). It was worth noticing that the expansion of HER2.28ζ CAR-T cells soon reached a plateau at day 9 post-stimulation with irradiated SKOV3^PD-L1^ cells (Fig. 3c), indicating that HER2.28ζ CAR-T cells lacked the ability of sustained proliferation. We also found that PD-L1.BB CSR-T cells had mild but persistent expansion after antigen stimulation, but the underlying mechanism needs to be further investigated. Furthermore, we detected a significantly higher proportion of CD45RO^+^CCR7^+^ T_CM_ subset in HER2.28ζ/PD-L1.BB CAR-T cells, either in culture medium or MPE-supernatant environment (Fig. 3d). Overall, these data demonstrated that PD-L1.BB CSR engagement endowed HER2.28ζ CAR-T cells with significantly enhanced proliferation activity, increased T_CM_ cell phenotype, as well as more durable response to antigen-stimulation.

Furthermore, to answer whether PD-L1.BB CSR-modified CAR-T cells can overcome MPE/MA-mediated inhibition, we further evaluated the cytotoxicity and proliferation activity of HER2.28ζ/PD-L1.BB CAR-T cells in MPE-supernatant from a NSCLC patient (Pt1). Similar to that in culture medium, HER2.28ζ/PD-L1.BB CAR-T cells showed superior tumor-killing activity, and formed obviously larger cell clusters when co-incubated with SKOV3^PD-L1^ cells for 24 h, as compared to HER2.28ζ CAR-T cells and PD-L1.BB CSR-T cells (Fig. 3e). Next, CAR-T and UTD cells were incubated with SKOV3^PD-L1^ cells in MPE-supernatant at different E:T ratios (1:2, 1:1, 2:1, and 4:1) for 24 h. As expected, the anti-tumor cytotoxicities of CAR-T cells were all remarkedly impaired compared to those in culture medium. Despite being functionally suppressed by MPE environment, HER2.28ζ/PD-L1.BB CAR-T cells exhibited significantly superior cytotoxicity compared to other CAR-T cell groups (Fig. 3f). To further characterize PD-L1.BB CSR incorporation-mediated proliferation enhancement, the absolute T-cell counts were detected by flow cytometer after stimulated with irradiated SKOV3^PD-L1^ cells in MPE-supernatant. The result showed that HER2.28ζ/PD-L1.BB CAR-T cells had sustained proliferation up to 9 days after antigen-stimulation, while the T-cell expansion in HER2.28ζ and PD-L1.BB groups were remarkably decreased after 3 to 5 days of co-culture with SKOV3^PD-L1^ cells (Fig. 3g).

Previously, we and others reported that long-term exposure to MPE/MA led to epithelial-mesenchymal transition (EMT), antigen loss, as well as treatment resistance.^16,31^ To exclude the potential influence of continuous exposure to MPE/MA, such as HER2 antigen loss, we cultured SKOV3^PD-L1^ cells in MA-supernatant from an OSC patient (Pt4) for 1 month (MA-treated SKOV3^PD-L1^), and found that the levels of HER2 and PD-L1 expressions were similar on MA-treated and untreated SKOV3^PD-L1^ cells (Fig. 2c and Supplementary Fig. S3). Although HER2.28ζ/PD-L1.BB and HER2.28ζ CAR-T cells were both activated when co-cultured with MA-treated or untreated SKOV3^PD-L1^ cells in MA-supernatant, we found that T-cells expressing HER2.28ζ/PD-L1.BB had significantly higher secretion of IFN-γ, IL-2 and TNF-α (Fig. 3h). To further evaluate the cytotoxicity to primary cancer cells, MPE was obtained from a small cell lung cancer (SCLC) patient (Pt10) and confirmed by pathologists (Supplementary Fig. S4), HER2 and PD-L1 expressions on cancer cells were detected by flow cytometry (Fig. 3i). Then co-culturing different CAR-T cells with enriched primary tumor cells from MPE. Despite of the low PD-L1 expression on primary cancer cells, enhanced tumor killing and cytokine release were observed for HER2.28ζ/PD-L1.BB CAR-T cells as compared to HER2.28ζ CAR-T cells (Fig. 3j, k). Together, these data strongly indicated that PD-L1.BB CSR engagement enhanced the fitness and functions of HER2.28ζ CAR-T cells in MPE/MA environment.

### PD-L1.BB CSR engagement enhances 4-1BB expression and impairs PD-1/PD-L1 signals

**Fig. 4:**
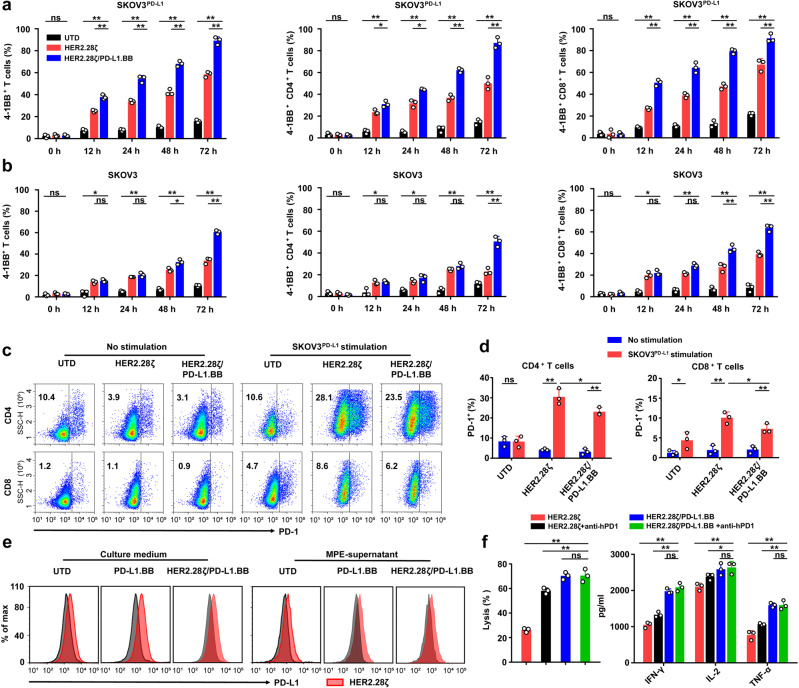
PD-L1.BB CSR engagement enhances 4-1BB expression and inhibits PD-1/PD-L1signals. **a**, **b** Statistical analysis of 4-1BB expression on HER2.28ζ and HER2.28ζ/PD-L1.BB CAR-T cells after stimulation. CAR-T cells were stimulated by either **a** irradiated SKOV3^PD-L1^ or **b** SKOV3 cells (E: T = 2: 1) in culture medium, and 4-1BB expression was assessed by flow cytometry at the indicated time points. **c** Representative flow cytometry plots showed PD-1 expression on CD4^+^ and CD8^+^ CAR-T cells on day 5 after stimulation (E: T = 2: 1). **d** Statistical analysis of PD-1 expression on CD4^+^ and CD8^+^ CAR-T cells. **e** PD-L1 expression on CAR-T cells. CAR-T cells were incubated with irradiated SKOV3^PD-L1^ cells (E: T = 2: 1) for 5 days, either in culture medium or MPE-supernatant (Pt1, as Supplementary Table. S1). **f** The cytotoxicity and cytokine release of HER2.28ζ and HER2.28ζ/PD-L1BB CAR-T cells before and after PD-1 blockade. CAR-T cells were stimulated with irradiated SKOV3^PD-L1^ cells, and treated with PD-1 blocking antibody (Tislelizumab, 10 μg/ml) for 3 days. Then the post-stimulated CAR-T cells were co-incubated with SKOV3^PD-L1^ (E: T = 2: 1) for 24 h. The cytotoxic activity of CAR-T cells was analyzed by CCK-8, and effector cytokine (IFN-γ, IL-2 and TNF-α) in culture supernatant were measured by ELISA. *P*-values were determined by one-way ANOVA with Tukey’s multiple comparison test adjusted *P* value (**a**, **b**, **f**) or unpaired two-tailed t-test **d**. *P <0.05; **P <0.01, ns, not significant. Data show the mean ± SD from three independent experiments

As the key co-stimulatory receptor on T-cells, 4-1BB is rapidly upregulated upon TCR signaling, and can reflect the proliferative capacity of activated T-cells.^26,28^ According to the design of PD-L1.BB CSR-modified dual-targeting CAR system, PD-L1 antigen-binding can result in the dimerization of 4-1BB intracellular domain, which potentially endows CAR-T cells with amplified 4-1BB signaling.^32^ Therefore, we detected 4-1BB expression on CAR-T cells, using an antibody specifically targeting its extracellular domain. The results showed after stimulation with irradiated SKOV3^PD-L1^ cells, 4-1BB was remarkably elevated on T-cells from all groups, while HER2.28ζ/PD-L1.BB CAR-T cells had a significantly higher expression as early as 12 h post-stimulation as compared to HER2.28ζ CAR-T cells, both on CD4^+^ and CD8^+^ T cell subsets, and lasted to at least 72 h (Fig. 4a). By contrast, when stimulated with irradiated SKOV3 cells that had moderate PD-L1 expression, this discrepancy was delayed to 48 h, which indicated that the upregulated 4-1BB expression was induced by PD-L1.BB CSR engagement (Fig. 4b). In addition, the activation of CD28 or 4-1BB signaling upregulates T-cell exhaustion markers including PD-1 and TIM-3.^26^ Notably, we found that after antigen-stimulation, PD-1 expression was remarkably upregulated on both CD4^+^ and CD8^+^ T cell subsets of CAR-T cells. However, HER2.28ζ/PD-L1.BB CAR-T cells had significantly lower PD-1 expression than HER2.28ζ CAR-T cells (Fig. 4c, d), whereas no obvious difference was observed for TIM-3 (Supplementary Fig. S5). In addition, considering that PD-L1 expression on T-cells can be induced either by antigen-stimulation or MPE/MA,^18,20,33^ the PD-L1 expression on CAR-T cells was further evaluated. The results showed that after antigen-induced activation, HER2.28ζ CAR-T cells had higher PD-L1 expression than HER2.28ζ/PD-L1.BB CAR-T and PD-L1.BB CSR-T cells, no matter in culture medium or MPE environment (Fig. 4e). Owing to CAR-T cell activation-induced PD-1/PD-L1 upregulation, then PD-1 blockade was used in combination with CAR-T cells for solid tumor treatment.^7,27^ The efficacy of PD-1 inhibitor plus HER2.28ζ or HER2.28ζ/PD-L1.BB CAR-T cells were compared in vitro, using an anti-PD-1 monoclonal antibody (Tislelizumab, commercially available from Beigene), As a result, HER2.28ζ CAR-T cells showed significantly increased cytotoxicity and release of effector cytokines (IFN-γ, IL-2, and TNF-α) after PD-1 blockade (Fig. 4f). By contrast, no significant changes were observed for HER2.28ζ/PD-L1.BB CAR-T cells, indicating the PD-1/PD-L1 axis was effectively blocked in PD-L1.BB CSR-modified CAR-T cells. Therefore, our data demonstrated that PD-L1.BB CSR engagement promotes amplification of 4-1BB signaling and blocked PD-1/PD-L1 axis-mediated immunosuppression, which finally developed activation and proliferation advantages for HER2.28ζ/PD-L1.BB CAR-T cells.

### PD-L1.BB CSR engagement-mediated rapid and persistent eradication of pleural and peritoneal metastasis

**Fig. 5:**
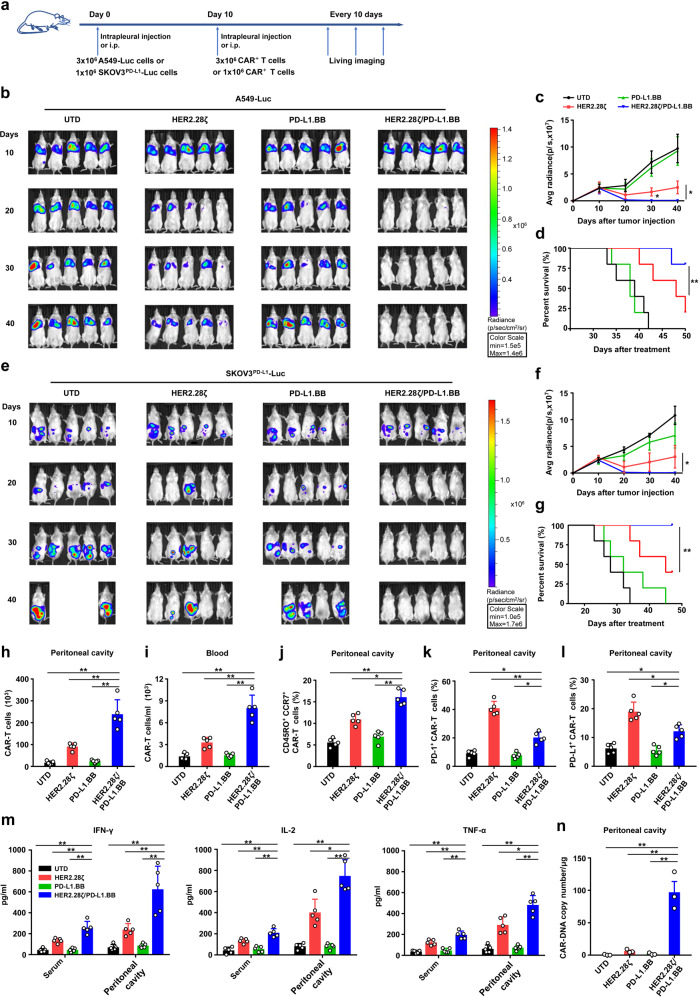
HER2.28ζ/PD-L1.BB CAR-T cells have prolonged antitumor activity in vivo, with rapid and long-lasting eradication of pleural and peritoneal metastasis. **a** The schematic diagram of A549 and SKOV3^PD-L1^ metastatic xenograft models in NSG mice inoculated via intrapleural or intraperitoneal (i.p.) injection, and treated 10 days later with CAR-T cells. **b** Representative bioluminescence (BLI) images of A549-Luc tumor growth in the pleural metastasis xenograft model. Mice were intrapleurally given 3 × 10^6^ CAR^+^ T or UTD cells, and tumor progression was monitored by in vivo imaging system (IVIS). **c** The BLI kinetics of A549-Luc tumor growth in the pleural metastasis xenograft model (*n* = 5). **d** Kaplan–Meier survival curve of mice after CAR-T cell treatment (*n* = 5). **e** Representative BLI images of SKOV3^PDL1^-Luc tumor growth in the peritoneal metastasis xenograft model. Mice were intraperitoneally given 1 × 10^6^ CAR^+^ T or UTD cells, and tumor progression was monitored by IVIS. **f** The BLI kinetics of SKOV3^PDL1^-Luc tumor growth in the peritoneal metastasis xenograft model (*n* = 5). **g** Kaplan–Meier survival curve of mice after CAR-T cell treatment (*n* = 5). **h**, **i** Summary of CAR-T cell (CD45^+^CD3^+^) number in the peritoneal cavity **h** and peripheral blood **i** of SKOV3^PD-L1^-Luc peritoneal metastasis mice analyzed by flow cytometry 7 days after CAR-T cell treatment (*n* = 5). **j** The ratios of central memory T cells (CD45RO^+^CCR7^+^), PD-1^+^ **k** and PD-L1^+^ **l** CAR-T cells (*n* = 5). **m** The levels of effector cytokines (IFN-γ, IL-2 and TNF-α) in serum and peritoneal lavage fluid were measured by ELISA 7 days after CAR-T cells treatment (*n* = 5). **n** Quantification of CAR copy number by q-PCR to evaluate CAR-T cell persistence in the pleural cavity 2 weeks after SKOV3^PD-L1^-Luc tumor regression (*n* = 3). *P*-values were determined by one-way ANOVA with Tukey’s multiple comparison test adjusted *P* value (**c**, **f**, **h**, **i**, **j**, **k**, **l**, **m**, **n**) or long-rank test **d**, **g**. *P < 0.05, **P < 0.01, ns, not significant. Data are expressed as the mean ± SD of each group

To evaluate whether the incorporation of PD-L1.BB CSR enhances the antitumor activity of HER2.28ζ CAR-T cells in vivo, we implanted Fluc-transduced A549 and SKOV3^PD-L1^ cells into the pleural or peritoneal cavities of NSG mice, respectively. Ten days later, mice were infused with HER2.28ζ, PD-L1.BB, HER2.28ζ/PD-L1.BB CAR-T or UTD cells, either by intrapleural or intraperitoneal (i.p.) injection (Fig. 5a). In the A549 pleural metastasis model, HER2.28ζ/PD-L1.BB CAR-T cells rapidly eliminated pleural disseminated tumors within 10 days, and no tumor recurrence was observed for over 30 days after CAR-T cell treatment (Fig. 5b, c). In contrast, HER2.28ζ CAR-T cells showed incomplete elimination of disseminated tumors. On day 10 after CAR-T cell infusion, only 1/5 of HER2.28ζ CAR-T cell-treated mice had complete tumor remission, and tumor recurrence was observed 10 days later. In addition, the overall survival of HER2.28ζ/PD-L1.BB CAR-T cell-treated mice were significantly extended compared to HER2.28ζ CAR-T cell-treated mice (Fig. 5d). In the SKOV3^PD-L1^ peritoneal metastasis model, HER2.28ζ/PD-L1.BB CAR-T cells also exhibited superior tumor control, and peritoneal disseminated tumors were completely eradicated within 10 days. All mice remained tumor-free up to over 30 days post HER2.28ζ/PD-L1.BB CAR-T cell treatment, and the overall survival were significantly extended (Fig. 5e–g). In contrast, at 20 days after HER2.28ζ CAR-T cell treatment, only 3/5 of mice had complete tumor remission (Fig. 5e). Next, to evaluate whether the increased anti-tumor activity depend on the expression level of PD-L1 on target cell, we investigated the antitumor efficacy of HER2.28ζ/PD-L1.BB CAR-T cells in an abdominal metastasis model of parental SKOV3 cells with moderate PD-L1 expression as shown above. Expectedly, HER2.28ζ/PD-L1.BB CAR-T failed to eliminate the tumor completely, but still showed a better anti-tumor effect compared to HER2.28ζ CAR-T cells (Supplementary Fig. S6). This further supported that the inhibitory effect of PD-L1 was switched into activation signal by PD-L1.BB CSR, which was in line with the basic principle of our dual CAR design.

To further assess the persistence in vivo, CAR-T cells in the blood and abdominal cavity of SKOV3^PD-L1^ peritoneal metastasis mice were detected by flow cytometry after treatment. As a result, there were significantly more human CD45^+^CD3^+^ T cells in the peritoneal cavity of HER2.28ζ/PD-L1.BB CAR-T cell-treated mice on day 7 after CAR-T cell infusion, as compared to HER2.28ζ and PD-L1.BB CAR-T cell-treated counterparts (Fig. 5h), indicating a stronger proliferation of HER2.28ζ/PD-L1.BB CAR-T cells in vivo, while only a small number of CAR-T cells were detected in the circulating blood (Fig. 5i). Further investigation found a significantly increased percentage of CD45RO^+^CCR7^+^ T_CM_ cells in proliferating HER2.28ζ/PD-L1.BB CAR-T cells that isolated from the peritoneal lavage fluid (Fig. 5j and Supplementary Fig. S7a), which was consistent with the aforementioned findings in vitro (Fig. 3d). Interestingly, a slight but not significant increase of T_CM_ cell percentage was observed in PD-L1.BB CSR-T cell-treated mice, as compared to UTD cell-treated counterparts (Supplementary Fig. S8), suggesting the potential influence of PD-L1.BB CSR on T-cells.

Moreover, both PD-1 and PD-L1 expressions were significantly down-regulated on HER2.28ζ/PD-L1.BB CAR-T cells, as compared with HER2.28ζ CAR-T cells (Fig. 5k, l and Supplementary Fig. S7b, c), which further demonstrated that the PD-1/PD-L1 signaling pathway was blocked on CAR-T cells after PD-L1.BB CSR incorporation. In addition, the effector cytokines in peritoneal lavage fluids including IFN-γ, IL-2 and TNF-α were significantly increased in HER2.28ζ/PD-L1.BB CAR-T cell-treated mice (Fig. 5m), which has also been reported by others that the cytokine secretion of CAR-T cells was enhanced after PD-1/PD-L1 blockade.^34^ For long-term in vivo persistence, peritoneal lavage fluid was collected 2 weeks after tumor regression (day 34). Although CAR-T cells were hardly detected in most mice, a significantly higher CAR copy number was detected in HER2.28ζ/PD-L1.BB CAR-T cell-treated mice (Fig. 5n).

Overall, these data demonstrated that PD-L1.BB CSR engagement significantly enhanced the in vivo cytotoxicity, proliferation, and persistence of HER2.28ζ CAR-T cells, which finally leads to rapid and durable eradication of pleural and peritoneal metastasis.

### PD-L1.BB CSR engagement upregulates T-cell activation, cytotoxicity, and proliferation-related gene expressions

**Fig. 6:**
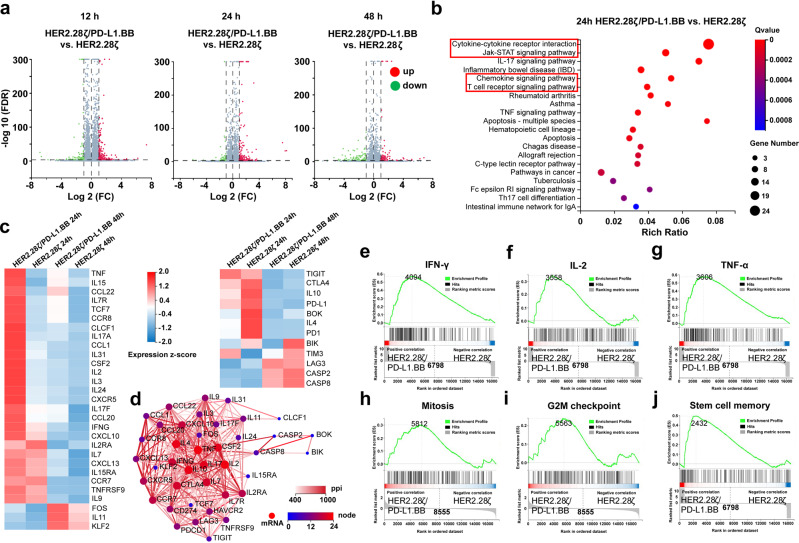
PD-L1.BB CSR engagement upregulates T-cell activation, cytotoxicity, and proliferation related gene expressions in HER2.28ζ/PD-L1.BB CAR-T cells. **a** RNA-seq analysis of HER2.28ζ/PD-L1.BB and HER2.28ζ CAR-T cells at 12 h, 24 h and 48 h after stimulation with irradiated SKOV3^PD-L1^ cells, and the differentially expressed genes (DEGs) were shown in volcano plot (FC ≥ 2, Q ≤ 0.05). FDR, false discovery rate; FC, fold change. **b** Significant enriched KEGG pathway terms of DEGs between HER2.28ζ/PD-L1.BB and HER2.28ζ CAR-T cells at 24 h post-stimulation (FC ≥ 2, Q ≤ 0.001). **c** Heatmap of selected DEGs with different expression related to cytokine-cytokine receptor interaction, chemokine and T cell receptor (TCR) signaling pathways. **d** Analysis of protein-protein interaction (PPI) networks of DEGs between HER2.28ζ/PD-L1.BB and HER2.28ζ CAR-T cells 24 h after stimulation. **e**–**j** Gene-set enrichment analysis (GSEA) of IFN-γ **e**, IL-2 **f**, TNF-α **g** signaling pathways, as well as mitosis genes **h**, G2M checkpoint genes **i** and stem cell memory pathway **j** was performed on all gene sets at 24 h post-stimulation. Data shown are representative of 2 replicates

To further elucidate the mechanism how PD-L1.BB CSR incorporation enhances the functions of HER2.28ζ CAR-T cells, transcriptome sequencing was performed at different time points post-stimulation with irradiated SKOV3^PD-L1^ cells. The results showed that HER2.28ζ/PD-L1.BB CAR-T cells had a different gene expression pattern compared to HER2.28ζ CAR-T cells at 12 h, 24 h, and 48 h post-stimulation. Total 493 differentially expressed genes (DEGs) were identified at 24 h (FC ≥ 2, Q ≤ 0.05), including 351 up-regulated genes and 142 down-regulated genes (Fig. 6a). We further identified 182 DEGs with more significant differences using stricter criteria (FC ≥ 2, Q ≤ 0.001), including 90 up-regulated genes and 92 down-regulated genes. KEGG enrichment analysis found that the majority of the 182 DEGs were enriched in cytokine, chemokine, Jak-STAT and T cell receptor (TCR) signaling pathways (Fig. 6b). HER2.28ζ/PD-L1.BB CAR-T cells had a distinct DEG profile compared to HER2.28ζ CAR-T cells after antigen-stimulation (Fig. 6c). Furthermore, the protein-protein interaction (PPI) network analysis showed that the main DEGs identified had close interactions, and the top 6 molecules were IL-10, IL-4, IL-2, IL-17A, IFN-γ and TNF-α. Among which, the expressions of anti-tumor cytokines including IL-2, IL-15 IL-17A, IFN-γ and TNF-α were up-regulated, while tumor-promoting cytokines including IL-4 and IL-10 were down-regulated in HER2.28ζ/PD-L1.BB CAR-T cells (Fig. 6d).

Meanwhile, downregulations of PD-1 and PD-L1 were observed in HER2.28ζ/PD-L1.BB CAR-T cells at transcriptome level, which was consistent with the protein expression level previously described (Fig. 5k, l). In addition, gene-set enrichment analysis (GSEA) performed on T-cell function-related gene sets further confirmed the upregulation of IL-2, IFN-γ and TNF-α signaling-associated genes, as well as T-cell activation and TCR signaling-associated genes in HER2.28ζ/PD-L1.BB CAR-T cells (Fig. 6e-g and Supplementary Fig. S9a, b), which explained the enhanced cytotoxicity of HER2.28ζ CAR-T cells after PD-L1.BB CSR incorporation. Moreover, GSEA showed that genes associated with cell cycle (mitosis and G2M checkpoint), as well as stem cell memory and effector memory pathways were upregulated in HER2.28ζ/PD-L1.BB CAR-T cells as compared to HER2.28ζ CAR-T cells (Fig. 6h–j and Supplementary Fig. S9c). Overall, the transcriptome analysis confirmed the incorporation of PD-L1.BB CSR up-regulated T-cell activation, cytotoxicity, and proliferation-related gene expressions in HER2.28ζ/PD-L1.BB CAR-T cells, and which was paralleled by the enhanced CAR-T cell functions and environment fitness presented above.

### The functional improvements of PD-L1.BB CSR-modified CAR-T cells are dependent on both PD-L1 scFv and 4-1BB domain

**Fig. 7:**
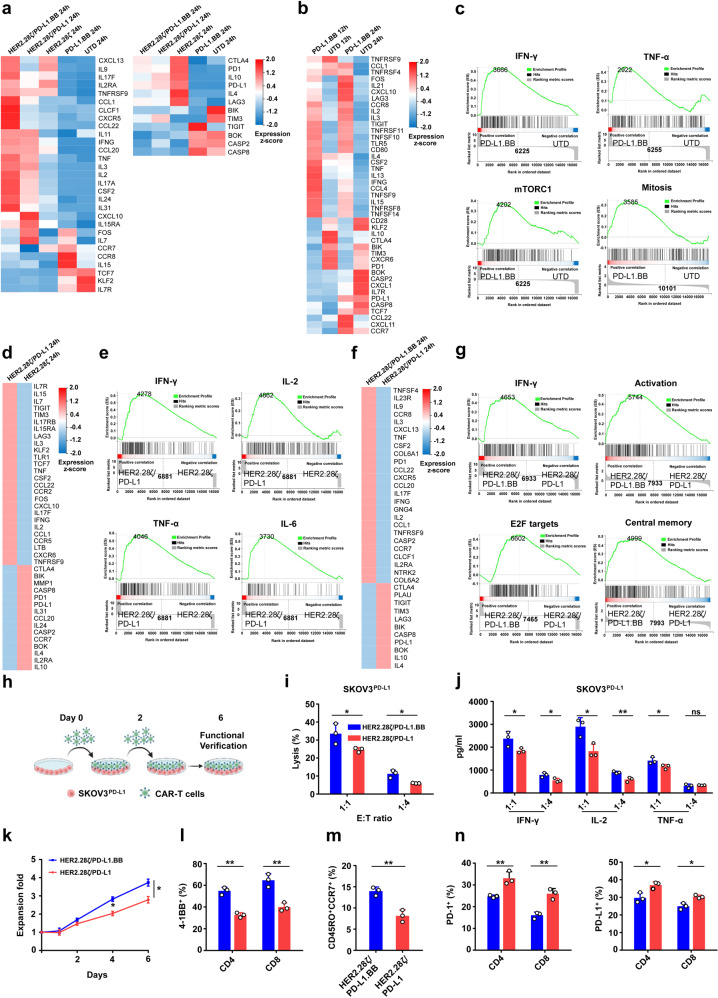
The roles of PD-L1 scFv and 4-1BB intracellular domain on the functional improvements of PD-L1.BB.CSR-modified CAR-T cells. **a** Heatmap of selected DEGs in HER2.28ζ/PD-L1.BB, HER2.28ζ/PD-L1, HER2.28ζ, PD-L1.BB CAR-T and UTD cells at 24 h post-stimulation with irradiated SKOV3^PD-L1^ cells. **b** Heatmap of selected DEGs between PD-L1.BB CSR-T and UTD cells at 12 h and 24 h post-stimulation. **c** GSEA of IFN-γ, TNF-α, and mTORC1 signaling pathway, as well as mitosis genes in PD-L1.BB CSR-T and UTD cells at 24 h post-stimulation. **d** Heatmap of DEGs between HER2.28ζ/PD-L1 and HER2.28ζ CAR-T cells at 24 h post-stimulation. **e** GSEA of IFN-γ, IL-2, TNF-α, IL-6 signaling pathways in HER2.28ζ/PD-L1 and HER2.28ζ CAR-T cells at 24 h post-stimulation. **f** Heatmap of selected DEGs between HER2.28ζ/PD-L1.BB and HER2.28ζ/PD-L1 CAR-T cells at 24 h post-stimulation. **g** GSEA of IFN-γ signaling pathway, T cell activation genes, E2F target genes and stem cell memory pathway in HER2.28ζ/PD-L1.BB and HER2.28ζ/PD-L1 CAR-T cells at 24 h post-stimulation. **h** The schematic diagram of repeated-stimulation experiment. At days 0 and 2, CAR-T cells were stimulated with irradiated SKOV3^PD-L1^ cells at E: T ratio of 2:1, and at day 6, CAR-T cells were sorted by human CD3 magnetic beads for functional test **i**, **j**, **m**, **n**. The cytotoxicity of post-stimulated CAR-T cells was analyzed by CCK-8, after 24 h of co-culture with SKOV3^PD-L1^ cells at indicated E: T ratios **i**, and effector cytokine (IFN-γ, IL-2 and TNF-α) secretion in culture supernatant was detected by ELISA **j**. **k** At days 1, 2, 4 and 6 of repeated-stimulation experiment, the proliferation folds of CAR-T cells were quantified by flow cytometry using absolute counting bead. **l** Statistical analysis of 4-1BB expression on CD4^+^ and CD8^+^ CAR-T cells on day 1 of repeated-stimulation experiment. **m, n** Statistical analysis of CD45RO^+^CCR7^+^ T_CM_ cells **m**, PD-1^+^ and PD-L1^+^ expressions on CD4^+^ and CD8^+^ CAR-T cells **n** at day 6 of repeated-stimulation experiment. Representative RNA-seq pictures shown in 2 replicates. *P*-values were determined by unpaired two-tailed t-test **i**–**n**. *P <0.05, **P <0.01, ns, not significant. Data are expressed as the mean ± SD from three independent experiments

In order to further answer whether PD-L1 scFv and 4-1BB, the two important components of PD-L1.BB CSR, are both necessary for functional optimization of HER2.28ζ/PD-L1.BB CAR-T cells, the gene expression profiles between different CAR-T groups and UTD cells were analyzed at 12 h and 24 h post-stimulation with irradiated SKOV3^PD-L1^ cells. Most selected genes were derived from the DEGs that identified between HER2.28ζ/PD-L1.BB and HER2.28ζ CAR-T cells as previously described (Fig. 6c), and obvious differences were observed among CAR-T cell groups (Fig. 7a). In addition, GSEA was performed to further assess the changes of T cell-related gene sets. Firstly, as compared to UTD cells, T-cell activation-related cytokine gene expressions including CSF-2, IL-2, IFN-γ and TNF-α, were significantly up-regulated in PD-L1.BB CSR-T cells, and downregulation of CTLA-4, PD-1 and TIM-3 genes were observed (Fig. 7b). Although PD-L1.BB CSR-T cells showed no significant cytotoxicity or effector cytokine secretion compared to UTD cells (Fig. 2d–g), GSEA revealed elevated IFN-γ signaling-associated genes at the transcriptome level. Meanwhile, only mild upregulation of TNF-α and IL-2 signaling-associated genes was observed (Fig. 7c and Supplementary Fig. S10a). In addition, PD-L1.BB CSR-T cells showed upregulation of mTORC1 signaling- and mitosis-associated gene expressions (Fig. 7c), which might be consistent with the in vitro mild proliferation previously described (Fig. 3c, g). Interestingly, we observed an enrichment of T cell memory-associated genes (Supplementary Fig. S10b, c), however, only a slight increase in central memory phenotype was observed for PD-L1.BB CSR-T cells in vivo (Supplementary Fig. S8c). Together, these data suggested that in the absence of antigenic stimulus-mediated CD3ζ activation signal, the activation of PD-L1.BB CSR only caused mild increase in T-cell proliferation, despite alterations in gene expression were observed.

Next, to explore the potential influence of PD-L1 scFv on T-cell functions, a HER2.28ζ/PD-L1 CAR was designed (Supplementary Fig. S11a), which was similar in structure to HER2.28ζ/PD-L1.BB, but lacked the 4-1BB co-stimulatory domain. And the gene expression patterns were compared between HER2.28ζ/PD-L1 and HER2.28ζ CAR-T cells post-stimulation. As a result, the expressions of effector cytokines (IL-2, IL-15 IFN-γ, TNF-α) and chemokines (CCL1, CCL22) were more prominent in HER2.28ζ/PD-L1 CAR-T cells as compared to HER2.28ζ CAR-T cells (Fig. 7d). Consistent with this finding, GSEA revealed upregulation of IL-2, IFN-γ, TNF-α, and IL-6 signaling pathways-associated genes in HER2.28ζ/PD-L1 CAR-T cells (Fig. 7e). In addition, no significant enrichment in cell cycle- and T cell memory-associated genes were observed, indicating PD-L1 scFv expression on T-cell surface only caused enhanced T-cell activation-related cytokine signaling. To further investigate whether the addition of 4-1BB domain at the downstream of PD-L1 scFv affects the antitumor activity of HER2.28ζ/PD-L1 CAR-T cells, the cytotoxicity and cytokine release of HER2.28ζ/PD-L1.BB and HER2.28ζ/PD-L1 CAR-T cells were tested in vitro, but no significant difference was observed (Supplementary Fig. S11b, c), despite obvious differences were detected for cytokine genes including IL-2, IFN-γ, and TNF-α on mRNA level (Fig. 7f). However, GSEA revealed upregulation of T-cell activation and IFN-γ signaling-associated genes in HER2.28ζ/PD-L1.BB CAR-T cells, suggesting the addition of 4-1BB domain amplified cytokine-mediated activation signaling. Moreover, obvious enrichment of genes in cell cycle (E2F target and G2M checkpoint), central memory and stem cell memory T-cell differentiation was observed (Fig. 7g and Supplementary Fig. S12), which was consistent with the increased proliferation and T_CM_ cell generation of HER2.28ζ/PD-L1 CAR-T cells that previously described. Additionally, after repeated stimulation with irradiated SKOV3^PD-L1^ cells, HER2.28ζ/PD-L1.BB CAR-T cells continued to efficiently kill tumor cells (Fig. 7h, i), accompanied by significantly enhanced production of IFN-γ, IL-2 and TNF-α (Fig. 7j), as well as a stronger and more persistent proliferation, as compared to HER2.28ζ CAR-T cells (Fig. 7k). Meanwhile, a significantly higher 4-1BB expression and CD45RO^+^CCR7^+^ T_CM_ subset, as well as lower PD-1 and PD-L1 expressions were detected on HER2.28ζ/PD-L1.BB CAR-T cells (Fig. 7l–n).

**Fig. 8:**
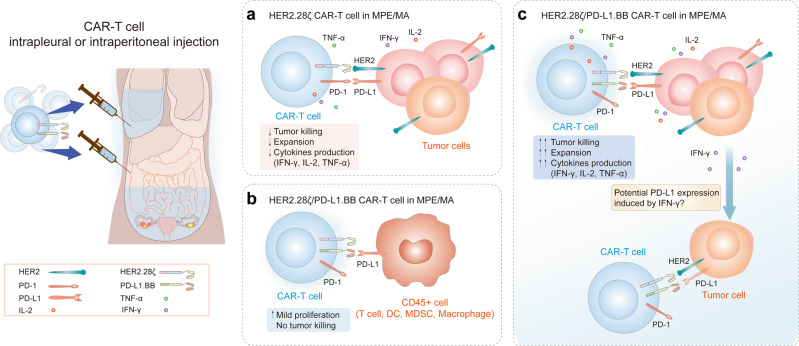
A virtual treatment scenario of CAR-T cells in pleural/peritoneal cavity. **a**–**c** The therapeutic pattern and potential mechanism of regional delivery of HER2.28ζ **a** and HER2.28ζ/PD-L1.BB CAR-T cells **b**, **c** to treat pleural or peritoneal metastasis accompanied by MPE/MA

Overall, we demonstrated that PD-L1.BB CSR plays an important role in the signal optimization of dual-targeting CAR system. The binding of anti-PD-L1 scFv to PD-L1 antigen blocks the inhibitory signal on CAR-T cells, and furtherly enhanced the production of effector cytokines. Meanwhile, the incorporation of 4-1BB intracellular domain at the downstream of PD-L1 scFv amplified T-cell activation signal, and more importantly, promotes the proliferation and T_CM_ phenotype generation of CAR-T cells. Based on the above concept and our findings, a virtual treatment scenario and possible mechanism of regionally delivered HER2.28ζ/PD-L1.BB CAR-T cells is shown in Fig. 8.

## Discussion

When developing CAR-T cell therapeutic strategies, particular attention should be paid to the environment-specific features. MPE/MA in serous cavities constitutes a localized, relatively closed liquid environment in which immune cells can direct contact with disseminated tumors, providing an opportunity for regionally delivered CAR-T cells to eliminate metastatic tumors. However, MPE/MA constitutes an immunosuppressive environment,^5,35^ which potentially induces CAR-T cell dysfunction. Here, we demonstrated that PD-L1.BB CSR-modification to a large extent rescued the functions of HER2.28ζ CAR-T cells, and the novel dual-targeting CAR-T cells exhibited superior fitness to serous cavity metastases and MPE/MA environment.

In the present study, we reported for the first time that T-cell activation-related cytokine release and proliferation of the 2nd-generation CAR-T cells were significantly inhibited by both the supernatant and cellular component of patient-derived MPE/MA. Based on these findings, there are two approaches to optimize CAR-T cell functions. The first one is to identify and block the key regulator of T-cell dysfunction, and the second one is to find and rescue the critical CAR-T cell functions that are being suppressed. However, the serous cavity environment formed by tumor metastasis is unique compared with that of solid tumors, which is frequently filled with malignant effusions that contain abundant immunosuppressive soluble factors and cells,^15–19^ so it is difficult to find the key inhibitory factor in this complex environment. Therefore, here we choose the second way to optimize CAR signals.

It is widely accepted that after CAR activation, the cytokine release and proliferation of CAR-T cells are highly dependent on the costimulatory signal provided.^26,36^ In the current study, we first demonstrated that when co-cultured with A549 cells, HER2.28ζ CAR-T cells displayed superior cytotoxicity compared with HER2.BBζ CAR-T cells. Our finding was consistent with a recent study in which the CD28 co-stimulated CAR-T cells also exhibited superior effector function against solid tumors than their 4-1BB co-stimulated counterpart.^25^ Nevertheless, we found that the effector cytokine secretion and proliferation of HER2.28ζ CAR-T cells were significantly inhibited in MPE/MA. Therefore, we hypothesized that to rescue impaired HER2.28ζ CAR-T cell functions in MPE/MA environment, the priority should be to enhance the positive costimulatory signal in CAR-T cells.

Researches have shown that PD-L1 was expressed on the cell surface of both disseminated tumor cells and immune cells in MPE/MA,^18,21,37^ and the expression could be further up-regulated by chemotherapy and PD-1 inhibitor treatment.^21,38,39^ In this study, we further confirmed that PD-L1 was broadly expressed on freshly-isolated cells of patient-derived MPE/MA, including tumor cells, MDSCs, macrophages and T cells. In this situation, it seems to be necessary to block the PD-1/PD-L1 axis to enhance CAR-T cell functions. Therefore, the novel PD-L1.BB CSR was designed, which can switch the PD-L1 inhibitory signal into an additional 4-1BB co-stimulatory signal, and we demonstrated the incorporation of PD-L1.BB CSR significantly rescued HER2.28ζ CAR-T cell functions with enhanced cytotoxicity, cytokine release, and proliferation.

Dual-targeting CAR-T cells expressing two CAR constructs that targeting separate antigens and contain different co‐stimulatory domains have been reported with enhanced antitumor cytotoxicity.^27,40^ We verified that compared to the 2nd-generation counterpart, PD-L1.BB CSR engagement endows CAR-T cells with three advantages: enhanced activation-associated cytokine release, better proliferation capacity, and increased central memory phenotype. All the three characteristics are critical for the rapid and durable anti-tumor activity of CAR-T cells. Our finding was consistent with a study recently published by Dotti’s group,^40^ in which they reported that by simultaneously targeting two separate TAAs GD2 and B7-H3, dual-targeting CAR-T containing independently activating CD28 and 4-1BB pathways exhibited enhanced antitumor activity, and effectively prevented solid tumor escape via optimal co-stimulation and metabolic fitness. Unlike their design, we preferentially select PD-L1 as the second tumor antigen besides the tumor-killing antigen, because of the broad expression of PD-L1 in MPE/MA environment. Because of lacking CD3ζ signaling, the PD-L1.BB CSR only mediates PD-L1 signal conversion rather than tumor cell killing, which can avoid PD-L1.BB CSR activation-mediated cytotoxicity on PD-L1^+^ immune cells. Since the PD-1/PD-L1 axis inhibits the production of IL-2, IFN-γ and TNF-α, and impairs T-cell expansion,^41^ PD-L1.BB CSR plays dual-roles in blockade of PD-1/PD-L1 axis and mediating activation of 4-1BB. In addition, PD-L1.BB CSR-modified dual-targeting CAR-T cells may have better adaptability to serous cavity metastases environment, since 4-1BB co-stimulated CAR-T cells were reported with an increased metabolic fitness.^42^ Moreover, PD-L1 is induced by IFN-γ expression in tumor cells,^43^ which may potentially enhance the stimulation of PD-L1.BB CSR leading to stronger fitness and activity in return.

The success of immunotherapy targeting PD-1 or PD-L1 suggests that, even in the presence of specific cytotoxic T lymphocytes (CTL), immune checkpoint blockade remains critical for the eradication of tumors.^44^ As a unique type of CTL, CAR-T cell therapy combined with PD-1 inhibitor have shown improved efficacy in preclinical and clinical studies.^7,34^ In our study, the PD-L1.BB CSR not only blocks the PD-1/PD-L1 axis, but also switches the PD-L1 signal into 4-1BB signal and amplifies activation of HER2.28ζ CAR. Some groups also tried to transform PD-1 to a T-cell costimulatory receptor by replacing the transmembrane domain and cytoplasmic tail with the CD28 or 4-1BB intracellular costimulatory domain.^29,36^ However, these designs are unable to effectively prevent PD-L1/PD-1 binding on activated CAR-T cells. In the design of PD-L1.BB CSR, anti-PD-L1 scFv competes with CAR-T cell-expressed PD-1 for binding to PD-L1 since the binding affinity of antibody-antigen is much stronger than that of ligand-receptor and PD-L1 antibody can effectively block binding site of PD-1.^45^ Therefore, PD-L1.BB CSR can preferentially bind to PD-L1 of target cell via its scFv and avoid activation of PD-1 signal in CAR-T cell itself. Under the circumstances, it may not be necessary for combination of anti-PD-(L)1 or anti-4-1BB antibodies to enhance the efficacy of PD-L1.BB CSR-modified CAR-T cells. As shown above, tislelizumab failed to enhance the cytotoxicity of HER2.28ζ/PD-L1.BB CAR-T cells. Moreover, the transcriptomic and flow cytometry data showed that after CAR activation, HER2.28ζ/PD-L1.BB CAR-T cells had a significantly lower PD-1 expression than HER2.28ζ CAR-T cells, which may be explained by increased IFN-γ release.^46^ Meanwhile, PD-L1 was upregulated on HER2.28ζ CAR-T cells, and this may be attributed to antigen-mediated T-cell activation, which can further promote immune suppression as a consequence.^33^ However, HER2.28ζ/PD-L1.BB CAR-T cells showed decreased PD-L1 expression versus HER2.28ζ CAR-T cells. The decreased expressions of both PD-1 and PD-L1 are helpful for avoiding the interaction of CAR-T cells when activated.

In tumor environment, some immune cells that participate in anti-tumor immunity also express PD-L1 on the cell surface, including dendritic cells (DC), macrophages, and CD8^+^ T cells. ^47–49^ In our design, to avoid PD-L1 mediated cell killing, PD-L1.BB CSR uses a different transmembrane sequence from HER2.28ζ CAR and does not have CD3zata. The PD-L1.BB CSR can solely bind to PD-L1 positive target cells without HER2 antigen. According to the known mechanism of CAR binding and activation, we inferred the binding of PD-L1.BB CSR-T cell to the PD-L1 single positive cell would lead to homologous dimerization of 4-1BB intracellular domain, which is considered as a potential signal amplifier for 4-1BB signaling.^32^ An mildly increased proliferation of PD-L1.BB CSR-T cells was observed both in culture and MPE/MA, and PD-L1.BB CSR-T cells with a slightly higher proportion of central memory T cells in peritoneal lavage fluid. Furthermore, transcriptome sequencing revealed that DEGs were significantly enriched in IFN-γ signaling, mTORC1 pathway, and mitosis-related genes, among which mTORC1 activation has been reported to be important in T-cell proliferation.^50^ Unfortunately, we are unable to further investigate the potential in vivo interaction between PD-L1.BB CSR-modified CAR-T cells and other PD-L1^+^ cells besides tumor cells, due to the absence of PD-L1 expressing human stromal cells in immunodeficient mice. We speculate that PD-L1. BB CSR not only artificially increases the expression of 4-1BB domain in the absence of CD3ζ activation, but also causes consequent dimerization when binding to target cell via anti-PD-L1 scFv, further leading to 4-1BB domain aggregation and amplified activation. Indeed, we observed PD-L1.BB CSR engagement promoted enrichment of cell cycle-related genes, such as E2F targets, G2M checkpoint and mitosis, in HER2.28ζ/PD-L1.BB CAR-T cells, as compared to HER2.28ζ and HER2.28ζ/PD-L1 CAR-T cells. Notably, we observed significantly increased 4-1BB expression on activated HER2.28ζ/PD-L1.BB CAR-T cells, which was reported to be closely associated with persistence and increased central memory differentiation of CD8^+^ CAR-T cells.^42^ In fact, persistence and increased central memory T cells were observed both in vitro and in vivo. Therefore, the 4-1BB domain of PD-L1.BB CSR also played a very important role in function improvement for the dual-targeting CAR-T cells.

There was also an important finding that cannot be ignored that MPE/MA-supernatant exhibited stronger inhibition on CAR-T cell functions than MPE/MA-cells. We and others have demonstrated the presence of multiple inhibitors including TGF-β, IL-4, IL-10, and VEGF in MPE/MA.^15–17^ It is not clear which factor plays a major role in CAR-T cell function inhibition. The strategies using blocking or switching receptors targeting IL-4 or TGF-β may be also helpful for CAR design to treat pleural or peritoneal dissemination.^8,36^ For example, TGF-β targeting-based optimal strategies, including a TGF-β dominant-negative receptor, a TGF-β receptor (TGF-βR)-4-1BB chimera and a TGF-β-responsive CAR, have shown very good prospects.^8^ However, considering CAR-T cell activation and many of the immune checkpoints are initiated by receptor-mediating cell-to-cell interactions, we tend to use PD-L1 or other membrane antigens, as a target to design a synthetic receptor. Nevertheless, which design is more suitable for the treatment of pleural or peritoneal metastatic disease may require further confirmation via clinical trials. A better understanding of the effusion or ascites environment will help tailor more effective immunotherapies.

In summary, in the current study, we designed a novel PD-L1.BB CSR-modified dual-targeting CAR-T cell system, which addressed the challenges of immune inhibitory pleural and peritoneal metastasis environment accompanied by MPE/MA. This therapeutic strategy significantly enhanced CAR-T cell fitness and demonstrated great potential for clinical translation. Since tumor cells and PD-L1-expressing tumor-associated immune cells can be observed in a variety of tumor microenvironments,^37,51,52^ the current dual-targeting CAR design may provide a broad solution for CAR-T cell therapy strategies in solid tumors.

## Materials and methods

### Clinical specimens and ethics

This investigation was approved by the Ethics Committee on Biomedical Research, West China Hospital, Sichuan University. All methods involved were carried out in accordance with the Declaration of Helsinki. Human peripheral blood of healthy donors, malignant pleural effusion and ascites of cancer patients were collected, with informed consent waived. In total, 11 patients with MPE, and 13 patients with MA were enrolled (Supplementary Table. S1). Immediately following drainage, MPE/MA samples were transported on ice, and centrifuged at 845 × *g* for 10 min. The cell-free supernatant was aliquoted, frozen at −80 °C after centrifugation, and cell pellet was suspended in X-VIVO 15 medium (Lonza), supplemented with 5% human AB-serum (Sigma-Aldrich).

### Cell lines and agents

Human ovarian cancer cell line SKOV3, lung cancer cell lines (A549 and H1975), breast cancer cell lines (MDA-MB-468), Burkitt lymphoma cell line Raji, Human Umbilical Vein Endothelial cell line HUVEC and human embryonic kidney epithelial HEK-293T cells were stored in the State Key Laboratory of Biotherapy of Sichuan University. SKOV3^PD-L1^, SKOV3^PD-L1^-Luc and A549-Luc cells were generated by lentivirus transduction. A549, A549-Luc, H1975 and Raji cells were cultured in RPMI 1640 medium (Gibco), while SKOV3, SKOV3^PD-L1^, SKOV3^PD-L1^-Luc, MDA-MB-468, HUVEC and HEK-293T were cultured in DMEM medium (Gibco). All media were supplemented with 10% fetal bovine serum (PAN^TM^-Seratach), 100 U/ml penicillin, and 100 μg/ml streptomycin (HyClone^TM^). Cells were incubated at 37 °C in a humid atmosphere with 5% CO_2_. The anti-PD-1 monoclonal antibody tislelizumab (BGB-A317, BeiGene) was used to block the PD-1/PD-L1 axis.

### Plasmid construction

HER2-specific second-generation CARs (HER2.28ζ and HER2.BBζ) were assembled using HER2- single-chain variable fragment (scFv) (Trastuzumab), CD8α hinge/transmembrane region, CD28 or 4-1BB co-stimulatory domain, and CD3ζ domain. ROR1- and CD19-specific second-generation CARs were generated similarly, using the ROR1- or CD19-specific scFv (R12, FMC63), and CD28 intracellular domain (ROR1.28ζ and CD19.28ζ). PD-L1.BB CSR was generated using the PD-L1-specific scFv (YW243), CD8α hinge/transmembrane region, and 4-1BB co-stimulatory domain. The dual CAR (HER2.28ζ/PD-L1.BB) contains two individual CARs (HER2.BBζ and PD-L1.BB) which were linked by the T2A self-cleaving peptide sequence. The HER2.28ζ/PD-L1 CAR was similar in structure to HER2.28ζ/PD-L1.BB CAR, instead without the 4-1BB co-stimulatory domain. All CAR constructs were synthesized and cloned into the lentivirus vector pWPXLd (Addgene).

### CAR-T cell manufacturing

For lentivirus package, CAR expression plasmid, together with pMD2.G and psPAX2 packaging plasmids (Addgene) were transfected into HEK-293T cells by HighGene transfection reagents (ABclonal). Lentiviral supernatant was harvested 48 h and 72 h after transfection. Lymphoprep™ (Axis-Shield) was used to isolate and purify peripheral blood mononuclear cells (PBMCs) by gradient centrifugation. Then CD3^+^ T cell populations were isolated by CD3 MicroBeads (Miltenyi Biotec) according to the manufacturer’s instruction, and culured in X-VIVO 15 medium, supplemented with 10% human AB serum, 10 mM N-acetyl-cysteine, 10 mM glutamine, and 10 mM MEM amino acid (all purchased from Sigma-Aldrich), as well as 100 IU/ml rhIL-2, 5 ng/ml rhIL-7, and 5 ng/ml rhIL-15 (all purchased from Novoprotein). After stimulation by Dynabeads^TM^ Human T-Expander CD3/CD28 (Gibco) for 24 h, virus was added to activated T cells in RetroNectin-coated plate and centrifuged for 2 h at 1000 × *g*. Twenty-four hours after transfection, T cells were replaced with fresh medium. 3 days after transduction, CAR expression was detected by flow cytometry.

### Flow cytometry assay

FITC-conjugated anti-human HER2 antibody (24D2, BioLegend), and APC-conjugated anti-human PD-L1 antibody (MIH2, BioLegend) were used to determine the expression levels of HER2 and PD-L1 on different cancer cell lines. FITC-conjugated recombinant human HER2 protein, PE-conjugated human PD-L1 protein and FITC-conjugated human ROR1 protein (all purchased from Novoprotein), FITC-conjugated human CD19 protein (ACROBiosystems), were used to detect CAR expressions. PerCP-Cy5.5-conjugated CD45 (2D1), APC-conjugated CD3 (SK7), FITC-conjugated CD4 (OKT4), PE-conjugated CD8 (SK1), BV510-conjugated CD45RO (UCHL1), BV421-conjugated CCR7 (G043H7), PE-conjugated CD11b (LM2), PerCP-conjugated CD14 (HCD14), FITC-conjugated CD19 (HIB19), APC-conjugated CD33 (WM53), APC-conjugated 4-1BB (4B4-1), PE-Cy7-conjugated PD-1 (A17188B), BV605-conjugated TIM-3 (F38-2E2) antibodies were all purchased from the BioLegend. Dead/live cells were gated out by Zombie NIRTM (BioLegend). The apoptosis of CAR-T cells was evaluated by Annexin V-FITC/PI double staining analyais (BD Pharmingen). These data were acquired with ACEA NovoCyte flow cytometer, and analyzed by NovoExpress software (ACEA Biosciences, version 1.2).

### Co-culture experiment

CAR-T cells were co-cultured with target cells in X-VIVO 15 medium (Lonza) or clinically-derived MPE/MA component at different E: T ratios (1: 2, 1: 1, 2: 1, or 4: 1). Cells were analyzed at designated time points to measure the residual tumor cells and T cells.

### Proliferation assay

CAR-T cells were labeled with carboxyfluorescein diacetate succinimidyl ester (CFSE) according to the manufacturer’s instruction (Cell Trace™ CFSE Cell Proliferation Kit, Invitrogen), and then stimulated with 12-Gy irradiated target cells (SKOV3^PD-L1^ or MDA-MB-468) at E:T ratio of 2:1. CFSE dilution was analyzed by flow cytometry (FITC channel). The absolute T cell number was quantified using CountBright^TM^ absolute counting beads (Thermo Fisher Scientific).

### CCK8 assay

Co-incubation of CAR-T cells with tumor cells at specified E: T ratios (1: 2, 1: 1, 2: 1, or 4: 1) in 200 μl X-VIVO 15 medium or clinically-derived MPE/MA component in 96-well plate. Target cell viability was measured via Cell Counting Kit 8 (Beyotime), using a ELx800 microplate Reader (Biotek Instruments Inc). The lysis of target cells was calculated by the following formula: target cell lysis (%) = (1- (mixed cell absorbance-effector cell absorbance-medium control)/(target cell absorbance- medium control)) × 100.

### Cytokine release assay

The levels of the cytokines in co-culture supernatant, mouse serum, and peritoneal cavity fluid were measured by human IFN-γ, IL-2 and TNF-α ELISA Kit (BioLegend) according to the manufacturer’s instructions.

### Real-time cytotoxicity assay

The continuous CAR-T cell cytotoxicity was monitored using an xCELLigene Real-Time Cell Analysis (RTCA) Multiple Plates (MP) system (Aglient Technoligies). The real time survival of target cells was measured on this platform. Briefly, a total of 5 × 10^3^ SKOV3^PD-L1^ cells and 100 μl culture medium were added in each well of a 96-well E-plate (ACEA Biosciences), and placed into the RTCA MP instrument for approximately 12–15 h in a cell incubater. Then CAR-T cells were added into the corresponding wells, and the cell index values were calculated by xCELLigence RTCA software 2.1.0.

### Mouse xenograft models

To establish the in vivo pleural and peritoneal metastasis models, eight-week old female NSG mice (NOD.CgPrkdcscid IL2rgtm1Wj1/SzJ, Beijing Biocytogen Co., Ltd) were intrapleural or intraperitoneal (i.p.) injected with 3 × 10^6^ A549-Luc or 1 × 10^6^ SKOV3^PD-L1^-Luc cells (day 0), respectively. In A549 pleural metastatic model, tumor-bearing mice were randomly divided into 4 groups (*n* = 5), and intrapleurally injected with 3 × 10^6^ CAR^+^ T or untransduced (UTD) T cells (day 10). In SKOV3^PD-L1^ peritoneal metastatic model, tumor-bearing mice were randomly divided into 4 groups (*n* = 5), and intraperitoneally injected with 1 × 10^6^ CAR^+^ T or UTD cells (day 10). Tumor burden was measured every ten days by bioluminescence (BLI) imaging through the IVIS (PerkinElmer) with Living Image software v4.3.1. All procedures were performed in accordance with the Animal Care and Use Committee of the Experimental Animal Research Center of Sichuan University. Establishment of tumor pleural cavity metastasis model: The hair on the chest of the mice was shaved and the bared skin was sterilized with 75% alcohol, holding the fur on the back of the mice with the left hand; and the mice were fixed on the sterile operation table with the abdomen down, holding the syringe in the right hand at 5 mm above the line connecting the xiphoid process and the midaxillary line, and inserted the needle 3 mm perpendicular to the mice chest wall, 50 μl of the cell suspension was injected into the pleural cavity slowly.

### Quantitative reverse transcription PCR (RT-qPCR)

To assess the human PD-L1 mRNA expression, MPE/MA cell pellets were separated by centrifugation. The extraction of RNA by the TIANGEN® Kit, reversely transcribed into cDNA (PrimeScript® 1st strand cDNA Synthesis Kit, TaKaRa), and quantitated by TB Green Premix ExTaq (TaKaRa) on QuantStudio real-time PCR system (BIO-RAD) according to the manufacturer’s instruction.

### Quantitative real-time PCR (q-PCR)

NSG mice were sacrificed 24 days after CAR-T cell treatment, injected i.p. with 5 ml ice-cold PBS to the abdominal cavity, and the peritoneal lavage fluid was collected. Genomic DNA of cells in the peritoneal lavage fluid was extracted using TIANGEN^®^ DNA Kit, the copy number of CAR gene was quantitatively analyzed using primers spanning the scFv on QuantStudio real-time PCR system (BIO-RAD).

### Transcriptome analysis

CAR-T cells were stimulated for 12 h, 24 h, and 48 h with 12-Gy irradiated SKOV3^PD-L1^ cells (E: T = 2:1) in X-VIVO 15 medium. Then CD3^+^ T cells were isolated by CD3 MicrobBeads (Miltenyi Biotec), and the extracted RNA was sent to the Microarray Core Facility of Beijing Genomics Institute (BGI) for RNA-seq analysis. Briefly, the mRNA library was constructed using Illumina mRNA Library kit and sequenced by the BGISEQ-500. RNA-seq data were aligned with HISAT (2.0.4). The R-DESeq2 package used to conduct the differentially expressed genes (DEGs) analysis. The DEGs with FC ≥ 2 and Q value ≤ 0.05 were presented by volcano maps. The genes with FC ≥ 2 and Q value ≤ 0.001 were selected to perform enrichment analysis using the Dr. Tom network platform of BGI (http://report.bgi.com). The protein–protein interaction (PPI) information was parsed from STRING database (STRING, v11). All heatmaps were presented by Z-score normalization method, and Gene-set enrichment analysis (GSEA) was performed on dataset using the MsigDB database: MsigDB C7 (immunologic signatures) and MsigDB H (Hallmark).

### Public single-cell RNA-seq (scRNA-seq) data analysis

Public scRNA-seq data of ovarian cancer ascites were downloaded from GSE146026. In total, 11000 single cells from 22 samples were profiled to understand the cellular diversity within malignant ascites by Smartseq2 and by 10x Genomics, and the statistical analysis of the relative expression of PD-L1 in tumor cell, fibroblasts, macrophages and T cell clusters shown.

### Statistical analysis

Data analyses were performed using GraphPad Prism 8 (GraphPad software version 8.0), and all data were shown as mean ± SD. Significant differences were analyzed by two-sided unpaired student-test, one-way ANOVA, or log-rank test. *P* < 0.05 was considered statistically significant.

## Supplementary Information

Supplementary Materials

## Data Availability

All research data of this article are available upon reasonable request by readers.
