# Supplementary material for: A PD-L1-targeting chimeric switch receptor enhances efficacy of CAR-T cell for pleural and peritoneal metastasis
Source: Signal Transduct Target Ther. 2022 Nov 19;7:380. doi: 10.1038/s41392-022-01198-2 (PMC9675732; doi:10.1038/s41392-022-01198-2)
Supplement: Supplementary file 1 — Supplementary Materials [file 41392_2022_1198_MOESM1_ESM.docx]

Supplementary Materials for

A PD-L1-targeting chimeric switch receptor enhances efficacy of CAR-T for pleural and peritoneal metastasis

Qizhi Ma^1a^, Xia He^2a^, Benxia Zhang^1a^, Fuchun Guo^1^, Xuejin Ou^1^, Qiyu Yang^1^, Pei Shu^1^, Yue Chen^1^, Kai Li^1^, Ge Gao^1^, Yajuan Zhu^3^, Diyuan Qin^1^, Jie Tang^1^, Xiaoyu Li^1^, Meng Jing^2^, Jian Zhao^1^, Zeming Mo^1^, Ning Liu^1^, Yao Zeng^1^, Kexun Zhou^3^, Mingyang Feng ^3^, Weiting Liao^3^, Wanting Lei^3^, Qiu Li^3^, Dan Li^4*^, Yongsheng Wang^1^^*^

^1^ Thoracic Oncology Ward, Cancer Center, and State Key Laboratory of Biotherapy, West China Hospital, Sichuan University, Chengdu, China.

^2^ State Key Laboratory of Biotherapy, West China Hospital, Sichuan University, Chengdu, China.

^3^ Cancer Center, West China Hospital, Sichuan University, Chengdu, China.

^4^ Institute of Respiratory Health, Frontiers Science Center for Disease-related Molecular Network, and Precision Medicine Research Center, Precision Medicine Key Laboratory of Sichuan Province, West China Hospital, Sichuan University, Chengdu, China

^a^ These authors contributed equally to this work.

Correspondence to: [lidan@wchscu.cn](mailto:lidan@wchscu.cn); wangys@scu.edu.cn

**This PDF file includes:**

Figures. S1 to S12

Table. S1


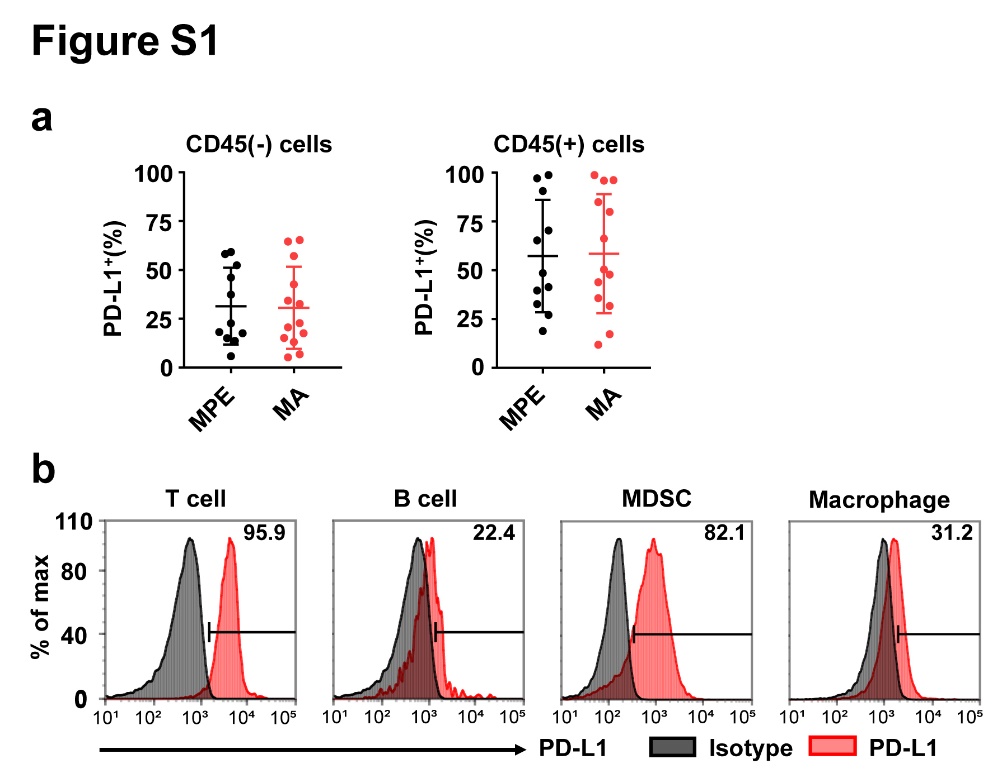


Figure. S1. The PD-L1 expression on MPE/MA cells.

**a** Statistical analysis of PD-L1 expression on CD45^+^ and CD45^-^ cells from freshly-isolated MPE (n=11) and MA (n=13) samples (Supplementary Table. S1). **b** Representative flow cytometry histograms of PD-L1 expression on T cells, B cells, MDSCs and macrophages of MPE sample (Pt1, as Supplementary Table. S1).


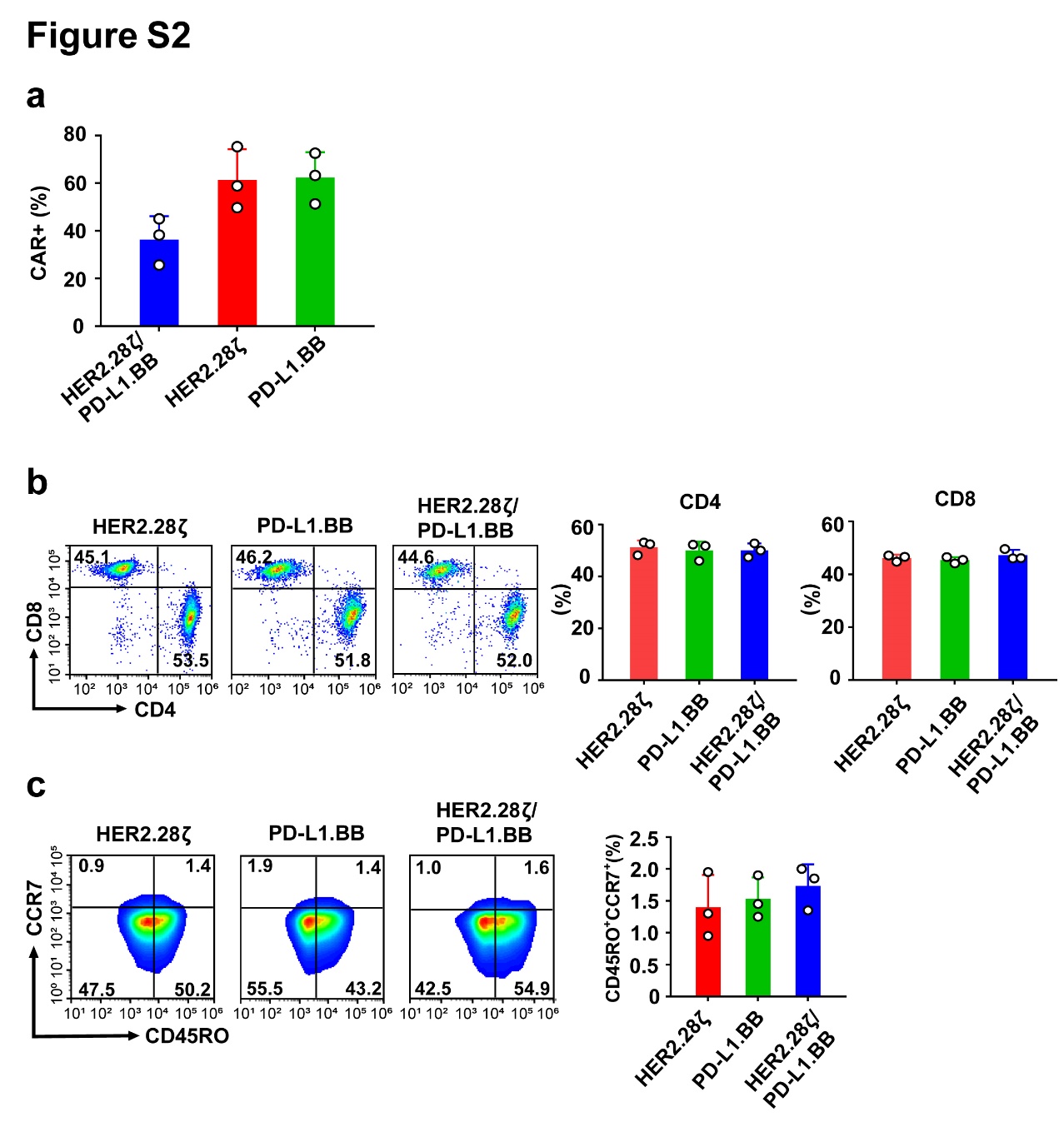


Figure. S2. CAR expression and phenotypic analysis of CAR-T cells.

**a** Statistical analysis of CAR expressions on healthy donor-derived T-cells that transduced with lentivirus encoding HER2.28ζ/PD-L1.BB, HER2.28ζ or PD-L1.BB. **b** The CD4^+^ and CD8^+^ subgroup ratios of CAR-T cells were detected by flow cytometry. **c** The ratios of central memory T cells (T_CM,_ CD45RO^+^CCR7^+^) in each group of CAR-T cells were analyzed by flow cytometry. Data shown the mean ± SD from three independent experiments.


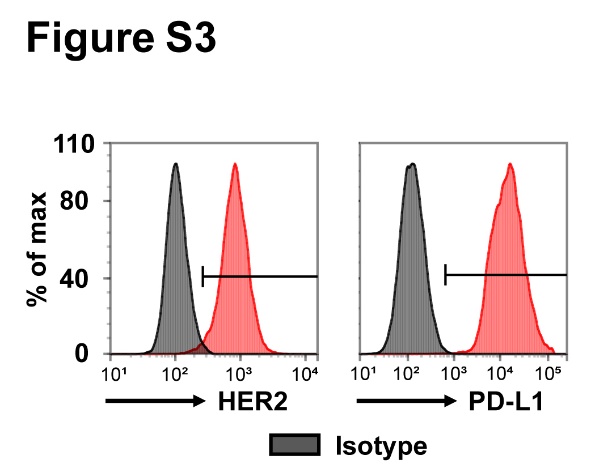


Figure. S3. HER2 and PD-L1 expressions on MA-treated SKOV3^PD-L1^ cells.

Representative flow cytometry histograms of HER2 and PD-L1 expressions on SKOV3^PD-L1^ cells that cultured continuously in MA-supernatant (Pt4, as Supplementary Table. S1) for 1 month (MA-treated SKOV3^PD-L1^).


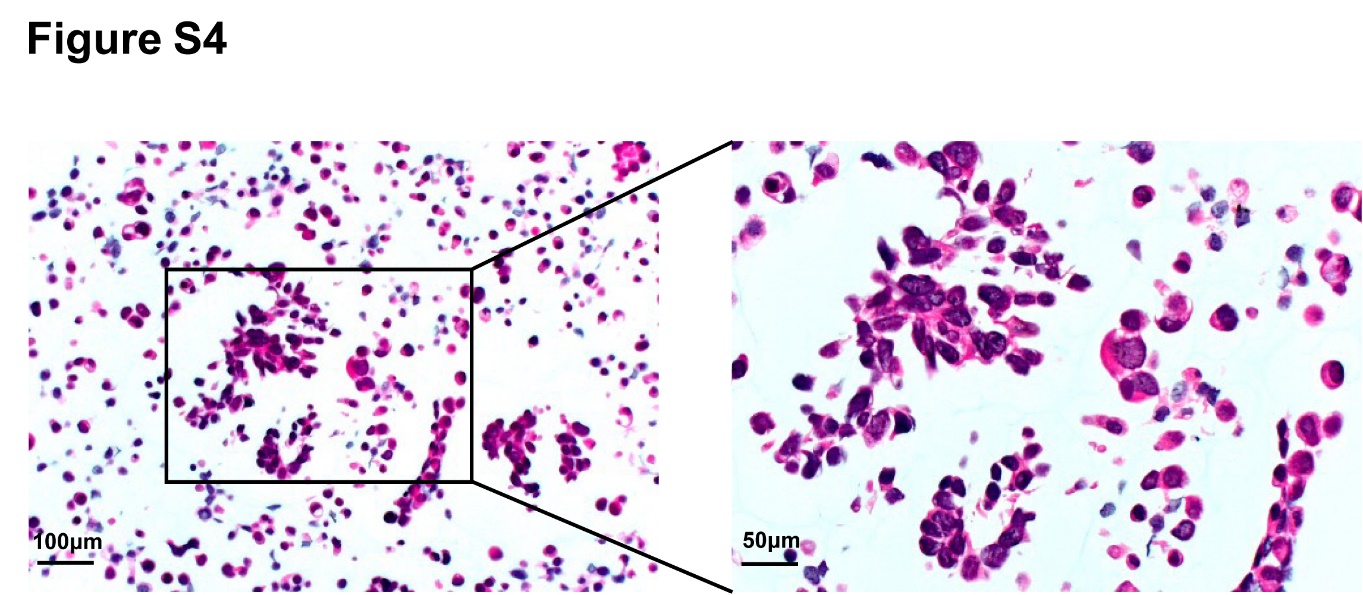


Figure. S4. H&E staining of exfoliated cells in MPE from a SCLC patient.

Representative microscopy images of exfoliated cells derived from MPE; collecting MPE derived a SCLC patient (Pt10, as Supplementary Table. S1), centrifuged to separate the cells. the scale bar represents 100 μm (left) and 50 μm (right).


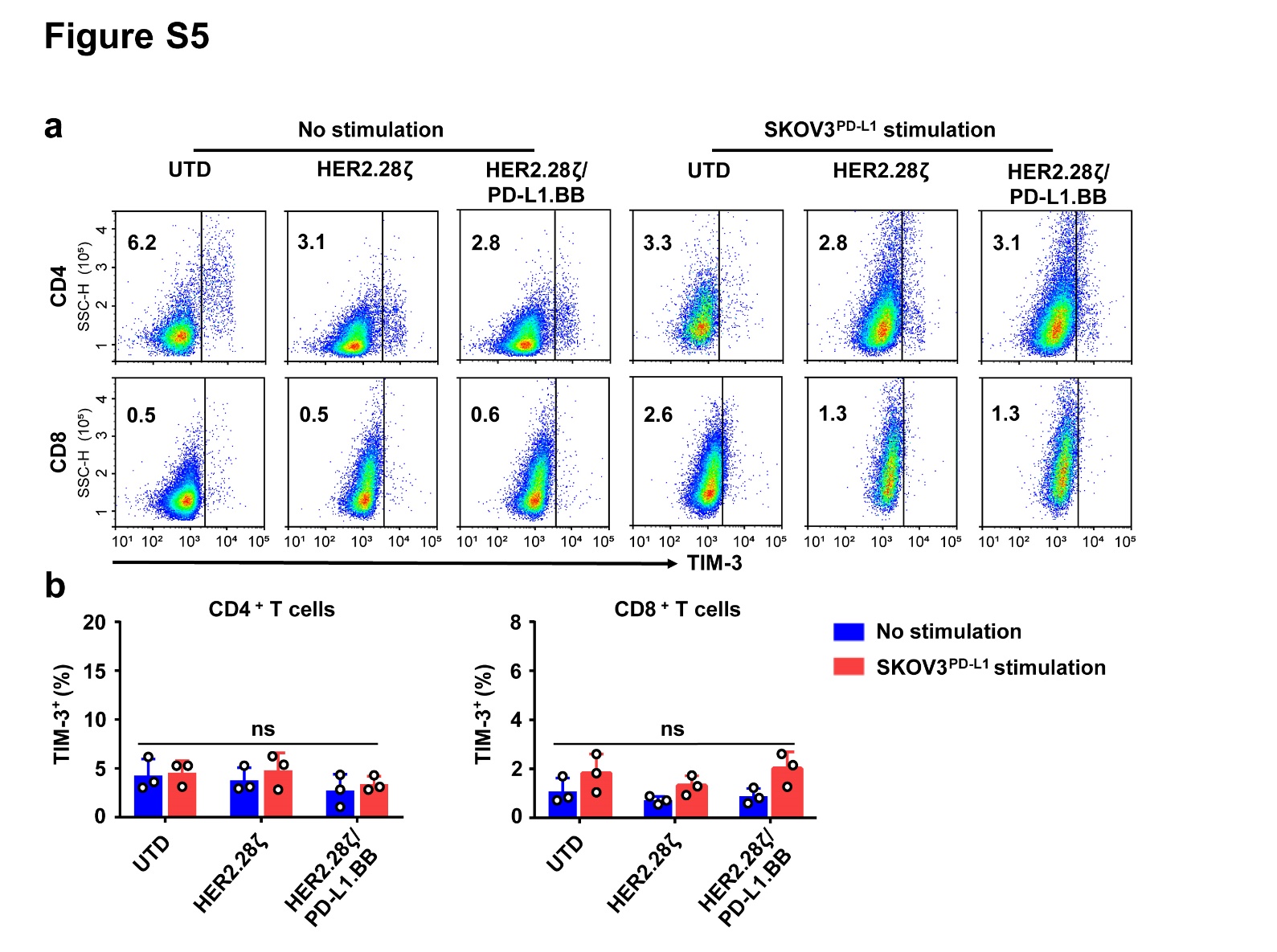


Figure. S5. TIM-3 expression on CAR-T cells.

**a** The expression of TIM-3 on CAR-T cells was detected by flow cytometry after 5 days of co-cultured with irradiated SKOV3^PD-L1^ cells at E: T ratio of 2:1. **b** Statistical analysis of TIM-3 expressions on CD4^+^ and CD8^+^ CAR-T cells. *P*-values were determined by unpaired two-tailed t-test (**b**). ns, not significant. Data show the mean ± SD from three independent experiments.


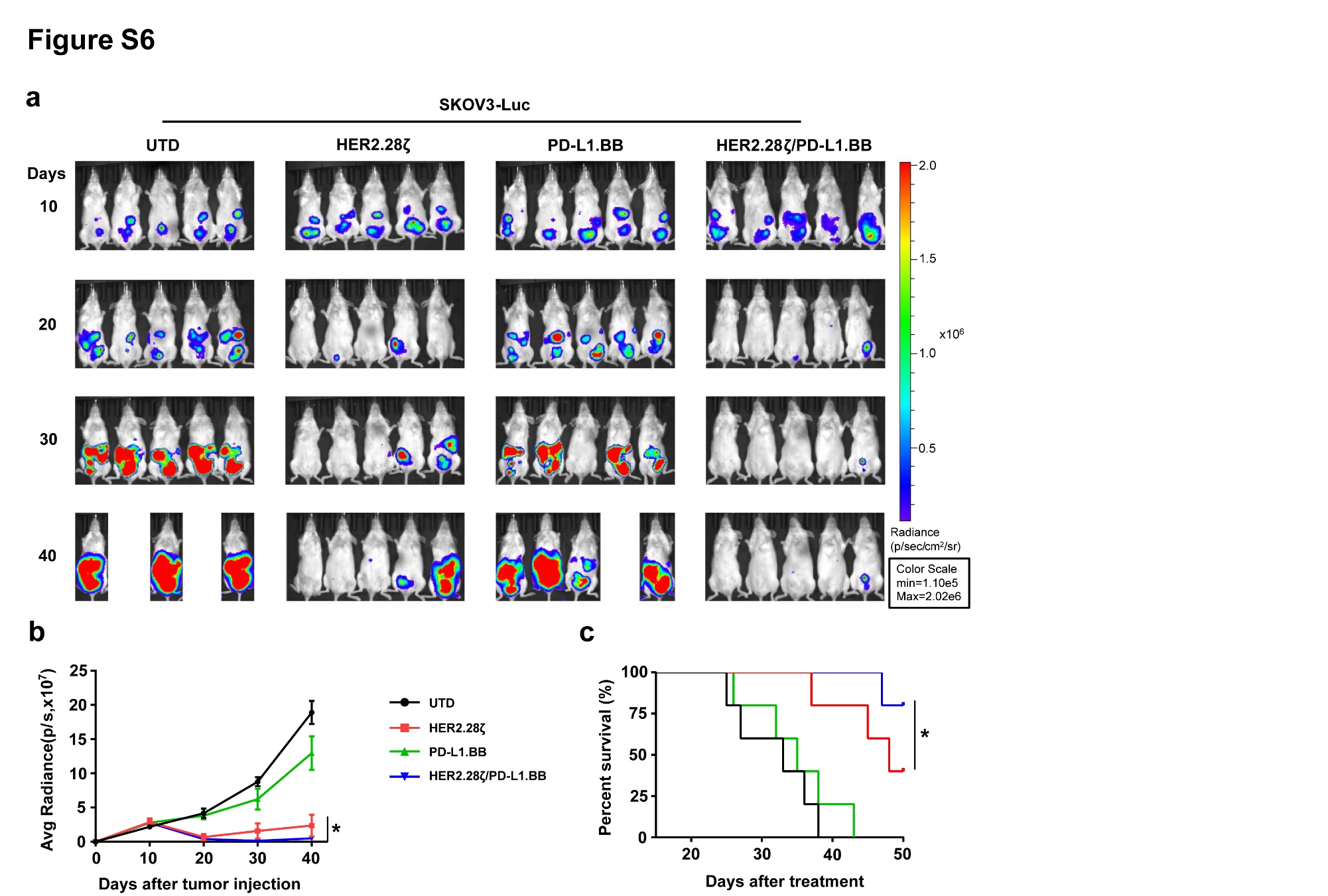


Figure. S6. HER2.28ζ/PD-L1.BB CAR-T cells rapidly and lastingly eradicate of SKOV3-Luc peritoneal metastasis with low PD-L1 expression *in vivo*.

**a, b** Representative tumor bioluminescence (BLI) images (**a**), and tumor BLI kinetics (**b**) of SKOV3-Luc tumor growth. NSG mice were injected intraperitoneally with 1 × 10^6^ SKOV3-Luc cells, 10 days later, the mice were treated with 1 × 10^6^ CAR^+^ T cells via intraperitoneal injection. The BLI signal indicating tumor progression by IVIS (n=5). **c** Kaplan-Meier survival curve of mice bearing SKOV3-Luc after CAR-T treatment (n=5). P-values were determined by one-way ANOVA with Tukey’s multiple comparison test adjusted P value (**b**) or Log-rank test (**c**). *P＜0.05.


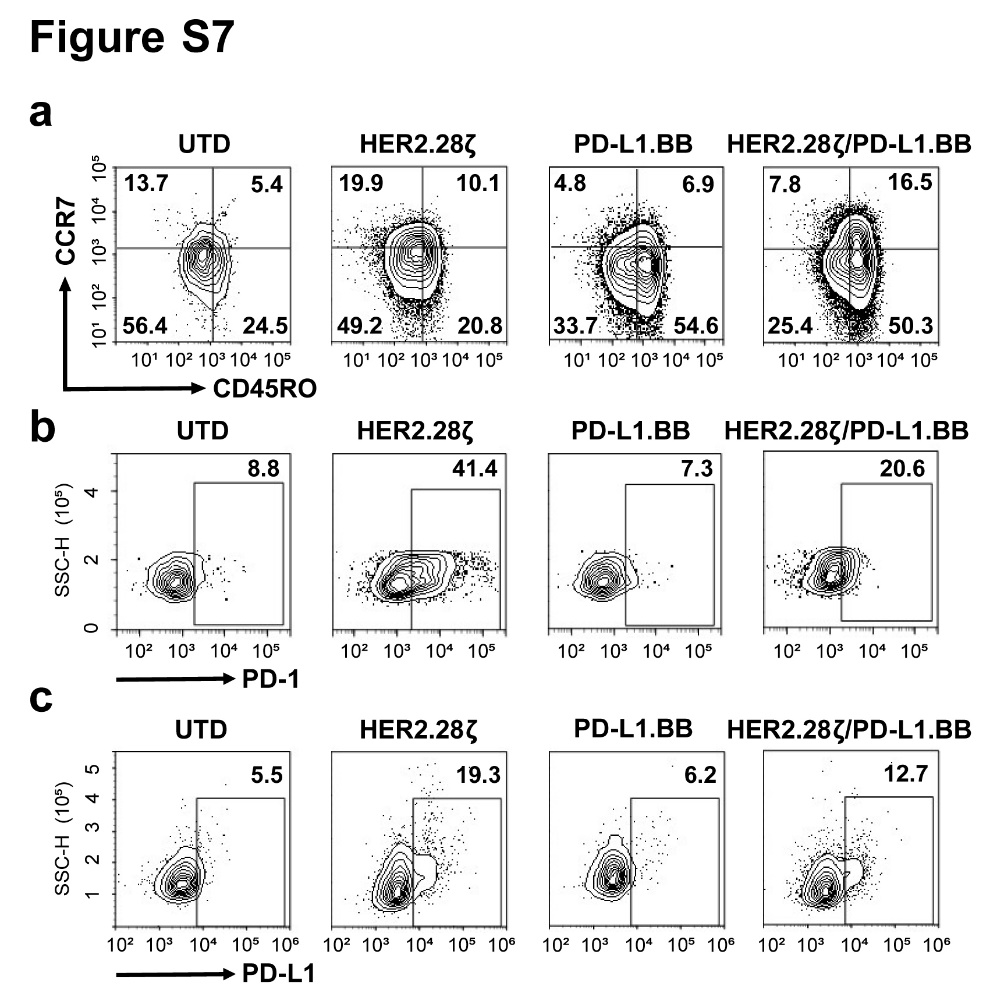


Figure. S7. PD-L1.BB CSR modification enhances the central memory phenotype and blocks PD-1/PD-L1 signaling pathway in HER2.28ζ/PD-L1.BB CAR-T cell *in vivo*.

**a** The representative flow cytometry plots of T_CM_ cells (CD45RO^+^CCR7^+^) in the peritoneal lavage fluid of SKOV3^PD-L1^-Luc peritoneal metastasis mice at 7 days after CAR-T cell inoculation. **b, c** The representative flow cytometry plots of PD-1 (**b**) and PD-L1(**c**) expressions on CAR-T cells (CD45^+^CD3^+^) in the peritoneal lavage fluid of SKOV3^PD-L1^-Luc peritoneal metastasis mice at 7 days after CAR-T cell inoculation.


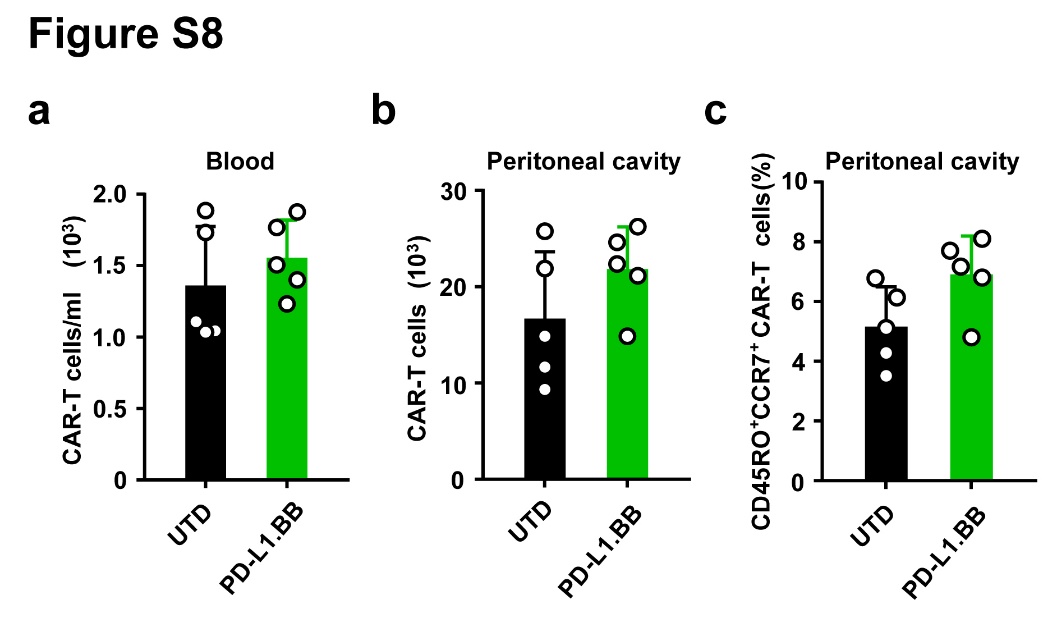


Figure. S8. PD-L1.BB CAR-T cells show a mild increase in T-cell expansion and central memory phenotype *in vivo*.

**a, b** Summary of CAR-T cells (CD45^+^CD3^+^) in the blood (**a**) and peritoneal cavity (**b**) of SKOV3^PD-L1^-Luc peritoneal metastasis mice at 7 days after CAR-T cell treatment (n=5). **c** Statistical analysis of T_CM_ (CD45RO^+^CCR7^+^) cells ratios in the peritoneal lavage fluid of SKOV3^PD-L1^-Luc peritoneal metastasis mice at 7 days after CAR-T cell inoculation (n=5).


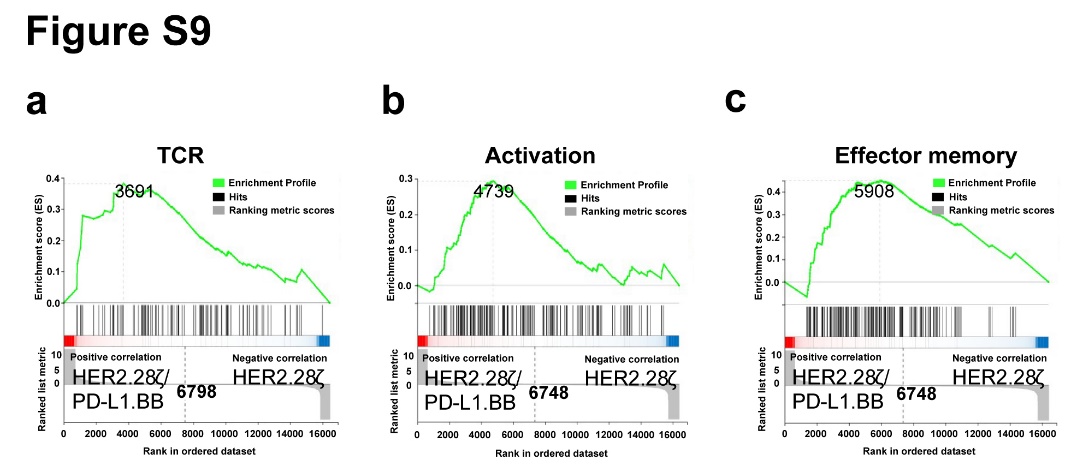


Figure. S9.

Gene-set enrichment analysis (GSEA) of TCR signaling pathway (**a**), T cell activation genes (**b**) and effector memory pathway (**c**) in HER2.28ζ/PD-L1.BB and HER2.28ζ CAR-T cells at 24 h post-stimulation.


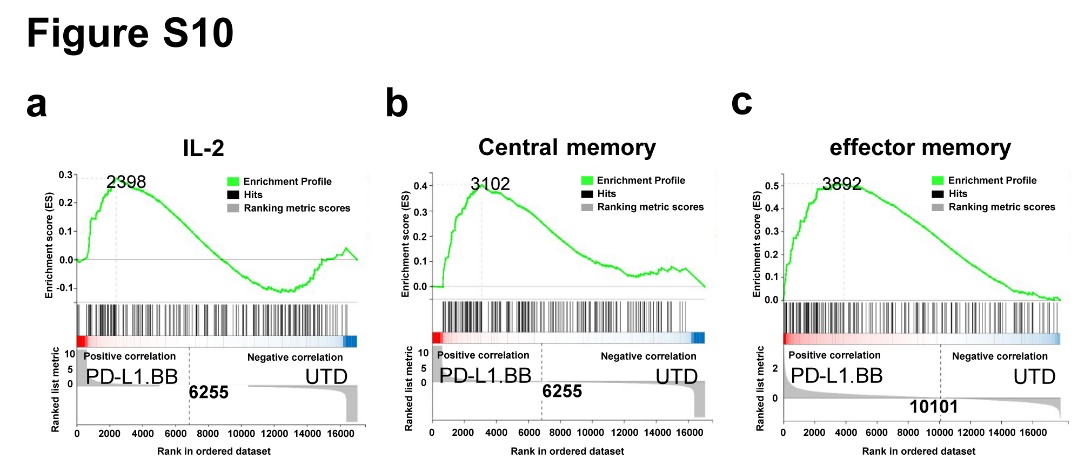


Figure. S10.

GSEA of IL-2 signaling (**a**), central memory (**b**) and effector memory pathways (**c**) in PD-L1.BB CAR-T and UTD cells at 24 h post-stimulation.


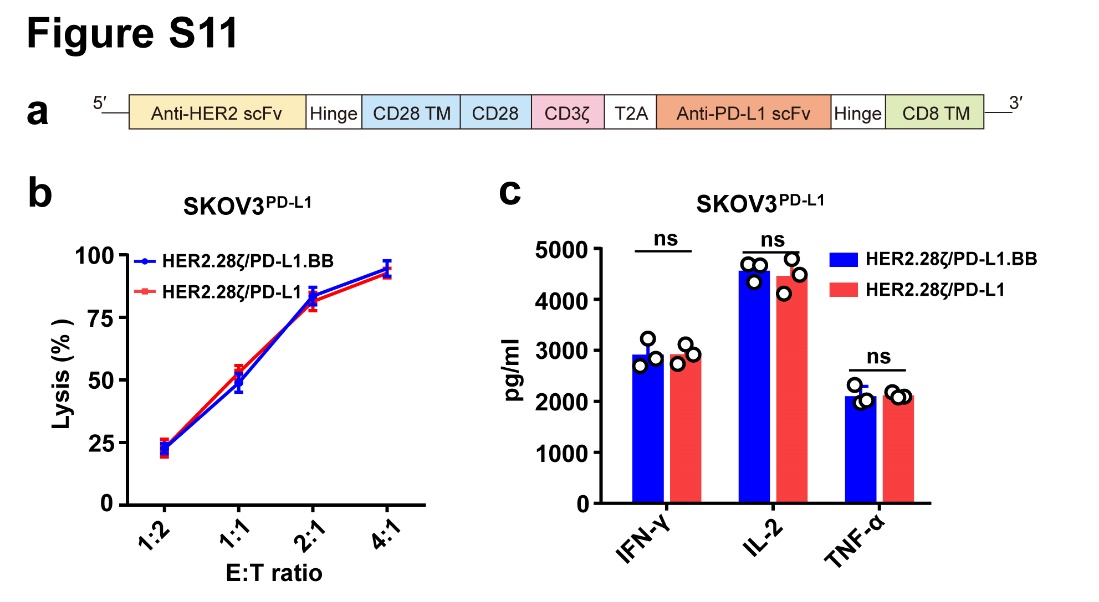


Figure. S11. The cytotoxicity and effector cytokine release of HER2.28ζ/PD-L1.BB and HER2.28ζ/PD-L1 CAR-T cells *in vitro*.

**a** Schematic illustration of lentiviral vectors encoding HER2.28ζ/PD-L1 CAR. **b** The effects of CAR-T cells cytotoxic activity were analyzed by CCK-8 assay; CAR-T cells were incubated with SKOV3^PD-L1^ in a cytokine-free medium for 24 h. **c** Summary of IFN-γ, IL-2 and TNF-α released by CAR-T cells in the culture supernatant after 24h of co-culturing with SKOV3^PD-L1^ (E: T = 2: 1) as measured by ELISA assay. P-values were determined by unpaired two-tailed t-test (**c**). ns, not significant. Data show the mean ± SD from three independent experiments.


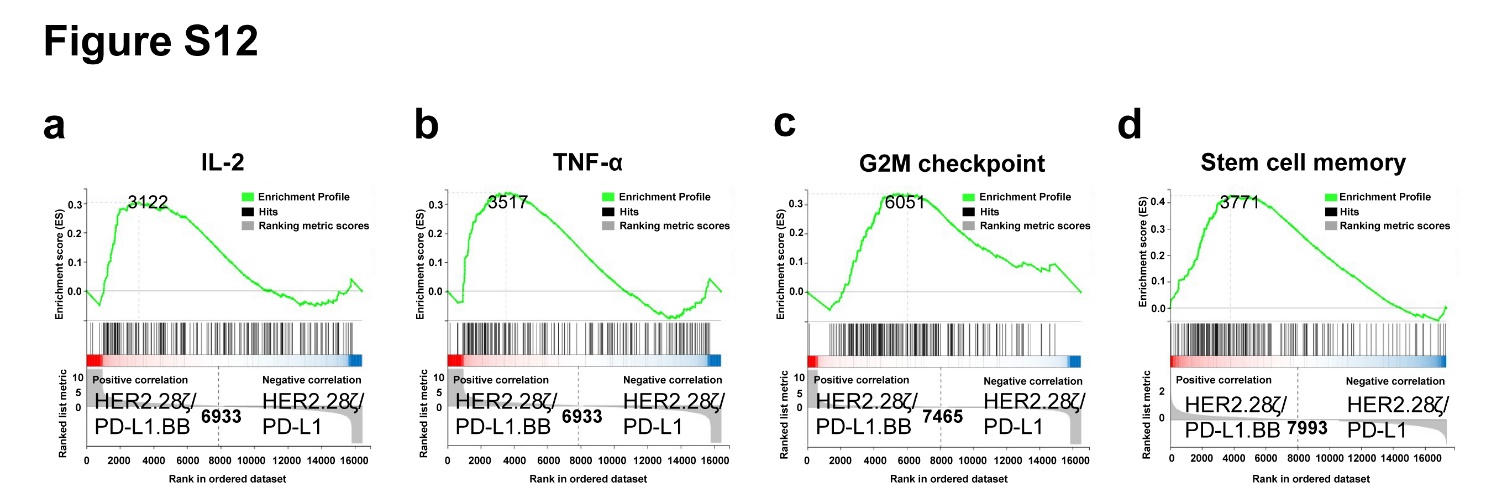


Figure. S12.

GSEA of IL-2 (**a**), TNF-α signaling pathway (**b**), G2M checkpoint (**c**) and stem cell memory genes (**d**) in HER2.28ζ/PD-L1.BB and HER2.28ζ/PD-L1 CAR-T cells at 24 h post-stimulation.

**Table. S1. Patient characteristics.**

| **Patient** | **Sex** | **Tumor origin** | **Effusion type** | **CD45^+^ (%)** | **Cell density (10^9^/L)** | **Experimental volume(ml)** |
| --- | --- | --- | --- | --- | --- | --- |
| 1 | M | NSCLC | MPE | 87.6 | 1.28 | 200 |
| 2 | F | NSCLC | MPE | 35.3 | 2.56 | 150 |
| 3 | M | NSCLC | MPE | 21.7 | 2.35 | 185 |
| 4 | F | OSC | MA | 56.3 | 1.71 | 350 |
| 5 | F | OSC | MA | 39.3 | 0.18 | 220 |
| 6 | F | OSC | MA | 40.4 | 0.31 | 110 |
| 7 | F | OSC | MA | 59.2 | 0.79 | 155 |
| 8 | M | PC | MA | 3.7 | 0.17 | 250 |
| 9 | M | NSCLC | MPE | 17.4 | 1.26 | 75 |
| 10 | F | SCLC | MPE | 51.3 | 1.03 | 145 |
| 11 | M | SCLC | MPE | 19.7 | 2.19 | 170 |
| 12 | M | GC | MA | 63.5 | 0.35 | 200 |
| 13 | F | GC | MPE | 7.8 | 0.56 | 280 |
| 14 | M | OSC | MA | 37.8 | 0.64 | 150 |
| 15 | F | NSCLC | MPE | 28.6 | 10.49 | 130 |
| 16 | M | SCLC | MPE | 31.9 | 2.14 | 80 |
| 17 | M | NSCLC | MPE | 5.1 | 12.33 | 200 |
| 18 | F | OSC | MA | 33.2 | 1.95 | 125 |
| 19 | F | FTC | MA | 11.4 | 0.43 | 240 |
| 20 | F | OSC | MA | 7.2 | 0.86 | 150 |
| 21 | M | PC | MA | 13.8 | 1.11 | 210 |
| 22 | M | NSCLC | MPE | 44.1 | 1.29 | 135 |
| 23 | F | OSC | MA | 52.4 | 0.29 | 200 |
| 24 | F | OSC | MA | 19.6 | 0.86 | 180 |

M, male; F, female; NSCLC, non-small-cell lung cancer; OSC, ovarian serous cystadenocarcinoma; PC, pancreatic cancer; SCLC, small-cell lung cancer; GC, gastric cancer; FTC, fallopian tube cancer; MPE, malignant pleural effusion; MA, malignant ascites.
